# Extracellular Vesicle-Based Therapeutics: Preclinical and Clinical Investigations

**DOI:** 10.3390/pharmaceutics12121171

**Published:** 2020-12-01

**Authors:** Natalia L. Klyachko, Camryn J. Arzt, Samuel M. Li, Olesia A. Gololobova, Elena V. Batrakova

**Affiliations:** 1 Center for Nanotechnology in Drug Delivery, University of North Carolina at Chapel Hill, Chapel Hill, NC 27599, USA; klyachko@email.unc.edu (N.L.K.); olgol@email.unc.edu (O.A.G.); 2 Eshelman School of Pharmacy, University of North Carolina at Chapel Hill, Chapel Hill, NC 27599, USA; camiarzt@live.unc.edu (C.J.A.); sammli@live.unc.edu (S.M.L.)

**Keywords:** drug delivery, extracellular vesicles, clinical applications

## Abstract

Drug nanoformulations hold remarkable promise for the efficient delivery of therapeutics to a disease site. Unfortunately, artificial nanocarriers, mostly liposomes and polymeric nanoparticles, show limited applications due to the unfavorable pharmacokinetics and rapid clearance from the blood circulation by the reticuloendothelial system (RES). Besides, many of them have high cytotoxicity, low biodegradability, and the inability to cross biological barriers, including the blood brain barrier. Extracellular vesicles (EVs) are novel candidates for drug delivery systems with high bioavailability, exceptional biocompatibility, and low immunogenicity. They provide a means for intercellular communication and the transmission of bioactive compounds to targeted tissues, cells, and organs. These features have made them increasingly attractive as a therapeutic platform in recent years. However, there are many obstacles to designing EV-based therapeutics. In this review, we will outline the main hurdles and limitations for therapeutic and clinical applications of drug loaded EV formulations and describe various attempts to solve these problems.

## 1. Introduction

Extracellular vesicles (EVs) have emerged as a new class of nanocarriers, triggering significant interest and enthusiasm. Extraordinary efforts have been made to develop new techniques that would make it possible to manufacture EV-based drug formulations for the treatment of various diseases, including cardiovascular diseases [1,2,3,4,5,6], regenerative disorders [7,8], infectious diseases [9], cancer [10,11,12,13,14,15,16,17,18,19], as well as autoimmune [20] and neurological disorders [17,21]. EVs are short- and long-distance mediators of intercellular communication that offer distinct advantages, uniquely positioning them as highly effective drug nanocarriers. They comprise various types of nanovesicles, including exosomes (30–120 nm), microvesicles (MVs) (50 nm–1μm), and apoptotic bodies (500–1000 nm) [22,23,24]. Notably, EVs consist of cellular membranes with multiple adhesive proteins on their surface [25,26] that enable efficient cell entry and delivery of therapeutic cargo.

The unique properties of EVs can be attributed to their biogenesis. Exosomes are initially produced by invagination of the endosomal membrane to create multivesicular bodies (MVB) [27]. In contrast, exosomes’ close relatives, MVs, are greater in size and bud directly from the plasma membrane. Therefore, exosomes and MVs originate from endosomal and plasma membranes, respectively. Apoptotic bodies form during the apoptotic process, when the cellular cytoskeleton breaks up, causing the membrane to bulge outward [28]. Different techniques have been developed for the characterization of EVs. Among them are nanoparticle tracking analysis (NTA) and dynamic light scattering (DLS), that provide information about: count (NTA) and size distribution (NTA and DLS); flow cytometry, western blotting, and mass spectrometry (MS) that can be used to characterize biochemical content of EVs; and several microscopy techniques Atomic Force Microscopy (AFM) and Cryogenic Transmission Electron Microscopy (CryTEM) that make it possible to assess EV morphology [29]. The structure, biogenesis and composition of EVs have been extensively described in several excellent reviews [7,18,21,30,31,32,33,34,35,36]. 

Similar to artificial nanocarriers, EVs can improve the fundamental characteristics of a free drug, such as its stability and solubility, and protect the drug against degradation in the bloodstream [31]. Relatively tight lipid bilayers in EV membranes can provide a sustained and prolonged release of the incorporated drug. Furthermore, contrary to most synthetic nanocarriers, EVs can cross biological barriers, including the blood brain barrier (BBB), making them especially valuable for the treatment of neurodegenerative disorders. It has been shown that EVs can cross the BBB from the brain to the bloodstream [37,38], as well as from blood to the CNS in vitro [39,40] and in vivo [41,42,43] under pathological conditions. However, whether EVs cross the BBB in the absence of pathology is still debated. Furthermore, these natural nanocarriers have low immunogenicity (especially, autologous EVs) and low cytotoxicity, which are usually substantial impediments for conventional synthetic nanoparticles. Finally, some types of EVs exert tissue tropism that makes it possible to target their formulations to specific cell types or migration towards inflamed tissues [44,45]. It is worth mentioning that bioinspired nanocarriers may have unique biological activity which is reflective of their origin, i.e., parent cells, that provides additional therapeutic efficacy to the incorporated drug [43]. These attractive features have contributed to the growing interest in EVs and inspired numerous studies aimed at their introduction to the field of drug delivery.

Despite these advantages, the clinical translation of EVs has been greatly slowed down due to a number of drawbacks, including upscaling processes of isolation and purification, as well as the lack of a means of efficiently loading these natural nanovesicles with therapeutics. Reliability, reproducibility, and donor-donor variations of EV formulations are still of significant concern. Furthermore, EV functional heterogenicity and limited yields represent serious obstacles for their future applications. Thus, depending on the mechanism of EV release, they may contain different proteins, active proteasomes, and even organelles (e.g., mitochondria) [46]. Inadequate targeting is another challenge for the clinical translation of different EV-based drug formulations. Herein, we will discuss how these hurdles can be overcome to introduce this unique biomimetic drug delivery system to the clinic.

## 2. Implications Related to Biological Activity Inherited from EV Origin

The biological activity of EVs released by various types of cells is vast and promising. Their ability to impact cells depends largely upon their protein markers and their cargo, which mimic the properties of their origin. Isolated EVs taken directly from specific types of cells, such as fibroblasts, neuronal cells, macrophages, and even cancer cells have a wide array of both pathogenic and therapeutic activities, largely depending their host cells. Therefore, one should pay special attention to the source of EVs and possible unwanted biological activity inherited from their parent cells. For example, EVs derived from diseased cells may contribute to the ability of a pathogen to spread throughout the body and evade the immune system [47]. Tumor-derived EVs are well-documented to express specific immune system markers such as MHC Class I and II molecules, death receptor ligands (FasL) and many others. The expression of these markers enables EVs to interact directly with prominent immune system cells such as T cells, B cells, and NK cells to encourage oncogenic activity and inhibit the immune system processes. Melanoma-derived EVs express FasL, which activates the Fas/FasL pathway to induce lymphocyte apoptosis, allowing tumors to evade cell-mediated cell death [47]. Next, EVs may contain prominent mediators that encourage angiogenic activity, metastasis, and mRNA transfer, leading to growth within the tumor microenvironment. Thus, gliomas, i.e., human brain and spinal cord tumors, express an oncogenic form of the epidermal growth factor receptor, EGFRvIII [48]. In mice, EVs containing EGFRvIII were shown to be released into the blood and fuse with tumor cells lacking EGFRvIII, conferring oncogenic activity upon previously benign cells. Moreover, cancer cell-derived EVs were shown to transport oncoproteins, including antigen MelanA/Mart-1 (melanoma), carcinoembryogenic antigen (CEA) (colon carcinoma), and HER2 (breast cancer) [49]. Finally, EVs can carry cancer-related miRNAs. Specifically, large amounts of small RNAs such as let-7, miR-1, miR-15, miR-16 and miR-375, which play an important role in cancer, were found in EVs [50]. Furthermore, Li et al. [51] studied the mechanism underlying the association between EVs and hypoxia during cancer progression. It was suggested that cancer cell-derived EVs mediate miRNA transfer and promote prometastatic behavior. Thus, oral squamous cell carcinoma (OSCC) cells secreted miR-21-rich EVs that ultimately contributed to the migration and invasion of OSCC cells [52]. In addition, miR-29a-3p carried by EVs from OSCC cells promoted M2-type macrophages polarization, and such macrophages enhanced the proliferation and migration of OSCC cells [53]. Hence, in many cases, it is preferable to use “clean” EVs without interior content that would not induce unwanted effects in patients. One approach to achieve this is to develop methods for the removal of the cargo of naive EVs without significant changes of the structure and content of their membranes. For example, Jang et al. [54] suggested using exosome-mimetic nanovesicles produced by the breakdown of monocytes via a serial extrusion through filters. These cell-derived nanovesicles should be depleted of their internal content inherited from parent cells.

Interestingly, EVs released by mesenchymal stem cells (MSCs) may deliver a bioactive cargo that inhibits or promotes tumor growth [55,56,57]. Thus, some studies indicated that MSC-derived EVs can play several roles in tumorigenesis, angiogenesis, and metastasis [42,58], although other studies showed tumor-suppressing effects [59,60,61,62]. These inconsistencies may be attributed to the source of parent MSCs, specifically, whether MSCs were obtained from cancer patients or healthy individuals [63]. Accordingly, nonmodified EVs may possess specific properties that would be beneficial to their therapeutic outcomes. For example, MSC-derived EVs have received much attention as potential therapeutic agents with regenerative properties [64,65,66,67,68,69,70], including protective effects in models of myocardial ischemia/reperfusion injury [65,66,71], pulmonary vascular disease [72], chronic myocardial infarction [73], and stroke [68,74,75,76]. Furthermore, EVs released by neural stem cells (NSCs) are known to promote neural tissue regeneration and functional recovery by releasing paracrine factors. In a recent report, Zhang et al. [77] demonstrated that the treatment of parent NSCs with interferon-gamma (IFN-γ) induced a generation of altered EVs that exerted improved therapeutic effects in an ischemic stroke rat model. Likewise, EVs derived from NSCs were shown to preserve and restore photoreceptors, decreasing apoptosis during retinal degeneration in rats [78]. Finally, EVs, particularly those produced by immune cells, are known to have immune-modulating, protective, and regenerative effects in conditions such as cardiovascular disease, atherosclerosis, and stroke [79]. Obviously, this additional biological activity may improve the therapeutic outcomes of drug-loaded formulations and should be considered when bio-inspired formulations are developed. For example, our earlier investigations demonstrated that naive EVs released by regenerative anti-inflammatory subtype of M2 macrophages produced synergistic neuroprotective effects in mouse models of Parkinson’s disease [43]. These effects were subtle but could be beneficial when added to the effects of incorporated therapeutics. Overall, these developments indicate that EVs can implement more than only inert carrier functions by being biological response modifiers. Further tailoring EVs may provide biologically active carriers that may be modified in accordance with the disease and produce, for example, the cytotoxic effects of EVs released by M1 macrophages for cancer treatment, or the neuroprotective effects of EVs released by M2 macrophages for the treatment of neurodegenerative disorders, and enhance the outcomes of their therapeutic cargo.

## 3. Improving Functional Heterogenicity and Yields of EVs Nanocarriers

EVs consist of various types of nanovesicles, namely exosomes, MVs, and apoptotic bodies [22,23,24]. It has shown to be difficult to separate EVs and MVs, mainly due to overlapping vesicle sizes and proteins expressed on their surface. Therefore, in most cases, a mixture of EVs and MVs is used to produce drug formulations [24]. It should also be noted that the absolute separation and definition of various EVs based on their size or biogenesis has yet to be established beyond doubt, and there is currently no consensus on markers that distinguish the origin of these vesicles once they have left the cell [80].

EVs can be isolated from conditioned cell culture media or bodily fluids by different methods, including differential centrifugation, filtration paired with centrifugation, concentration paired with ultracentrifugation, immunoaffinity chromatography, size exclusion chromatography, and polymer-based precipitation. Each isolation technique has advantages and disadvantages that should be considered in terms of being reproducible, specific, and feasible [81]. Differential ultracentrifugation combined with density gradient centrifugation are considered the “gold standard” for isolating EVs. This process involves applying a centrifugal force to a solution containing EVs, e.g., a conditioned cell culture media or biological fluids. It is worth noting that the type, quantity, and quality of EVs isolated by this method is sensitive to the *g* force, rotor type, angle of rotor sedimentation, radius of centrifugal force, pelleting efficiency, and solution viscosity. Gradient centrifugation requires extensive (62–90 h) centrifugation time [82], but provides a more uncontaminated EV isolate than ultracentrifugation alone. Of note, ultracentrifugation is associated with morphological alterations and partial aggregation of vesicles. Immunoaffinity chromatography is a more efficient method for isolating EVs as compared to differential ultracentrifugation and density gradient ultracentrifugation [81]. It requires a single easy step without using harsh chemicals. However, this method provides a relatively low yield and can be used for small volumes only. In addition, because this method of EV isolation depends on antibody recognition of EV proteins, only a subset of all EVs (those expressing the antibody-recognized protein) can be captured. Size exclusion chromatography (SEC) preserves the integrity and biological activity of EVs using gravity flow when vesicle structure and integrity remain intact. This is a fast and easy procedure that requires a small sample volume. However, a low concentrated sample needs an additional process for enrichment. Finally, polymer precipitation is relatively easy to use and does not require specialized equipment or a lengthy run time. However, it has been shown that this method coprecipitates nonvesicular contaminants such as lipoproteins, as well as polymer material [83]. Thus, Patel et al. [84] compared four EV isolation techniques for yield and purity. The polymer-based precipitation method had the maximum yield, followed by size-exclusion chromatography and differential ultracentrifugation. The immunoaffinity-based isolation method yielded the fewest EVs. Importantly, a high yield of EVs was accompanied by contaminations with serum proteins and chemical impurities, including high salt concentration, Sodium Dodecyl Sulfate (SDS), or Polyethylene glycol (PEG) contaminations after polymer-based precipitation. These issues may be addressed by pre- and post- isolation steps. Pre-isolation involves the removal of subcellular particles such as lipoproteins. Post-isolation involves removal of the polymer, typically by using a Sephadex G-25 column [82]. Therefore, considering large-scale clinical manufacturing [85], a level of segregation EVs from copurifying components may influence the functionality and therapeutic activity of the final product. 

The translation of EV-based formulations into clinical practice requires compliance with existing regulatory frameworks [86]. EVs are a fairly heterogeneous population in terms of their biochemical composition, size, and the source [87]. Thus, the standardization and effective purification of large amounts of these nanovesicles is a critical, but still considerable, challenge. Specifically, the manufacturing of homogeneous drug nanoformulations, and production and quality control, are crucial requirements. Moleirinho et al. [88] developed a purification method using semicontinuous multicolumn chromatography, a robust, scalable and efficient tool for EV purification. Besides the higher recoveries obtained with the continuous system when comparing with batch chromatography, the EV properties were maintained during the purification process regarding their size and morphology. A fast and reliable method of isolating serum EVs was reported by Navajas et al. [89]. Using size-exclusion chromatography with qEV columns (Izon, Christchurch, New Zealand), a homogeneous population of EVs in terms of size, morphology, and protein composition was obtained.

Another challenge that has critical implications for the use of EV-based formulations is whether the sufficient number of these carriers can be generated [90]. Indeed, the EV yield per cell will impact the final production cost, as well as having clinical applications. In this respect, the choice of parent cells is very important. For example, MSCs are known to produce large numbers of EVs, suggesting that these cells may be efficient for EV production in a clinically applicable scale [65,91]. Several reports have indicated that specific treatments of EV producing cells could considerably increase the yield of these natural nanocarriers. For example, culturing dendritic cells (DCs) for a prolonged time [92] or at low pH [93] increased EV production up to ten-fold. Furthermore, the addition of neutral and cationic-bare liposomes enhanced EV secretion in a dose-dependent manner [94]. However, the possible contamination of EVs with liposomes is a serious concern associated with this method. Gao et al. [95] reported high yield of EVs using nitrogen cavitation that instantly disrupted neutrophils to form nanosized membrane vesicles. The authors indicated that this approach made it possible to increase the manufacture of EVs by 16-fold. Another option is to break parent cells, for example, monocytes/macrophages with simultaneous loading with anticancer agents, followed by the isolation of EV-like nanoparticles [54]. In attempting to mimic the function of EVs with nanovesicles, Jang et al. [54] utilized human U937 monocytic cells to produce nanovesicles with the ability to carry large amounts of therapeutics. While maintaining the plasma membrane proteins of the targeted cells, the drug-loaded nanovesicles were able to efficiently induce tumor cell death and increase the production yield of chemotherapeutics in relation to naturally occurring EVs by 100-fold. Of note, one should consider that the alteration of cell culture conditions can certainly increase yield, but the impact on the biological effect of EVs has to be crucially assessed for biosafety reasons.

Next, the mass production of EVs by membrane fusion with lipid-based materials was suggested in several reports [96,97,98]. The manufacture of large quantities of drug nanocarriers was achieved via a membrane extrusion technique [96] that allowed up to a 43-fold increase in the numbers of vesicles postisolation. The production of hybrid EVs was also proposed by Rayamajhi et al. [97]. EVs from mouse macrophages were hybridized with synthetic liposomes that increased the yield and retention of the EV functional properties. The manufacture of hybrid EVs was also suggested by De La Peña et al. [98]. This group utilized coated liposomes as artificial EVs, and discovered that the obtained nanocarriers functioned as naturally occurring EVs and efficiently activated immune responses [98]. Chemically-induced membrane blebbing was suggested for the fast production of large numbers of EV-like vesicles [99]. Different chemical agents, for example, sulfhydryl, paraformaldehyde, or dithiothreitol, were shown to lock the cell in a fixed physiological state and promote the release of vesicles from a plasma membrane to the conditioned media. 

A different approach for the upscaling production of EVs was reported in a study conducted by Li et al. [100]. Instead of manufacturing EVs from animal cells, the authors utilized biocompatible bovine milk EVs (mEVs) that can be obtained inexpensively in large quantities [100]. mEVs were loaded with doxorubicin (Dox) and decorated with hyaluronan (HA), in order to direct them to CD44-overexpressing tumor cells. HA is a CD44-specific ligand which ensures that the EVs are directed to the cell membrane of the specified tumor cells. mEVs were able to deliver chemotherapeutics to tumor-specific cells in vitro and trigger apoptosis [100]. Crashed grapes were also suggested as an abounded source for EV-like nanoparticles [101]. Thus, the oral administration of EV-like nanovesicles from grapes facilitated intestinal regeneration in a mouse model of colitis that was induced by exposing mice to dextran sodium sulfate in drinking water. The EV-like nanovesicles prevented the colitis-associated reduction of both intestinal length and villus height. As a result, mice treated with grape-derived EV-like nanoparticles lived twice as long as untreated mice.

## 4. Loading EVs with Therapeutic Cargo

The closest relatives of natural nanocarriers EVs are synthetic vehicles, liposomes, and polymer-based nanoparticles. One of the major differences between these two drug delivery systems is that the loading of a drug into artificial vehicles can be achieved concurrently with their formation, when the drug is present at the time of nanoparticle formation. Contrary to synthetic-based formulations, therapeutics should be incorporated into EVs when they are already formed. It is worth noting that well-organized and tight EV membranes greatly restrict drug free penetration. In addition, substantial precautions should be made during drug loading to preserve the EV membrane structure, with all crucial proteins on their surface. This sets substantial restrictions on drug incorporation methods, and as a result, impedes the high loading efficacy of EV-based formulations. 

### 4.1. Exogenous Loading of EVs

Regarding exogenous loading, naive EVs isolated from parent cells media can be loaded with therapeutic agents by a variety of methods. These procedures include: co-incubation with a drug at different temperatures [41,43,54,102,103,104,105,106,107,108,109,110], sonication of EVs/drug mixture, including water bath sonication [44,97], and probe sonication [43,109,111,112,113]; electroporation of EVs with siRNA and miRNA [114,115,116], or EVs with small molecule anticancer drugs, paclitaxel (Ptx) and Dox [111]; precomplexation of siRNA with cationic liposomes followed by the fusion with isolated EVs [117]; transient permeabilization of EVs membranes with saponin for enzymes and other proteins [43,44]; freeze-thaw cycles [43]; or extrusion procedure [43]. Notably, aside from therapeutics, these methods can be applied to imaging agents. Specifically, magnetic resonance imaging (MRI) of EVs loaded with superparamagnetic iron oxide nanoparticles was suggested for tracking EVs to lymph nodes [118]. The main cases of different loading procedures are outlined in Table 1.

#### 4.1.1. Co-Incubation

Loading EVs with small molecule drugs by simple co-incubation can ultimately be utilized as a therapeutic treatment for a variety of diseases. Qu et al. [108] utilized this method of loading naive EVs isolated from mouse blood with dopamine by incubation with saturated solution of dopamine with 0.02% ascorbic acid for 24 h at room temperature (RT). The drug diffusion to the EVs luminal side was achieved via equalizing the concentrations. This novel drug delivery system was designed to allow the direct use of dopamine that would cross the BBB effectively with maximum biocompatibility and minimum toxicity in a mouse model of PD. The dopamine-loaded blood EVs were systemically injected into PD mice with acute brain inflammation caused by intracranial injection of 6-hydroxydopamine (6-OHDA). The encapsulation of dopamine in blood derived EVs significantly reduced its toxicity, and increased accumulation in the brain [108]. The authors suggested that blood EVs have a good natural targeting ability to the brain without any additional modification. Lipophilic small molecules, such as anticancer agents and anti-inflammatory agents, for example curcumin (Cur), can also be loaded by co-incubation with EVs. Sun et al. [41] reported that EVs from an EL-4 cell line were incubated with Cur at 22 °C, purified from nonincorporated drug, and injected intraperitoneally into mice with lipopolysaccharide (LPS)-induced septic shock. The results concluded that a significant reduction of inflammation was observed in LPS-intoxicated mice followed by treatment with Cur-incorporated EVs. Next, an anti-inflammatory drug, piceatannol, was loaded into neutrophil derived EVs by co-incubation via the pH gradient [95] using the same approach as was applied to Dox when loaded into liposomes [119]. Piceatannol is a weak acid molecule which increases the solubility in a basic environment. The pH gradient between the inner compartments of EVs (pH 9) and outside media (pH 4.5) made it possible to increase loading of piceatannol from 0.6% (drug(*w*)/vesicle(*w*)) with no pH gradient to 1.7% *w/w*. The authors reported that piceatannol-loaded EVs significantly alleviated acute lung inflammation/injury and sepsis induced by LPS. Co-incubation an acidophilic dye with a strong tumoricidal action, Acridine Orange (AO) with macrophage derived EVs was reported in [110]. AO charged EVs (EV-AO) showed an extended drug delivery time to melanoma cells as compared to free AO, improving drug cytotoxicity. Moreover, tumor reduction by EVs loaded with an anticancer agent, gemcitabine (Gem), was reported by Li et al. [109]. Thus, this chemotherapeutic drug has a history of significant side effects in the systemic treatment of pancreatic cancer. This off-site toxicity could be diminished by using EVs from the pancreatic cancer cell line Panc-1 as drug carriers. Gem-loaded EVs were able to suppress tumor growth or fully eliminate tumors in mouse model of pancreatic cancer.

The incorporation of high molecular weight therapeutics, such as protein and nucleic acids, into EVs has additional challenges related to the difficulty of penetration across the EV membranes for these large molecules, as well as their possible degradation during the loading procedure. A simple co-incubation with EVs at RT was also used for manufacture of EV-based formulation of a macromolecular therapeutic agent, brain-derived neurotrophic factor (BDNF) [45]. Macrophage-derived EVs were loaded with BDNF and intravenously injected into mice with brain inflammation [45]. It was reported that EVs were able to cross the BBB and deliver the protein cargo to the inflamed brain tissues. The increased brain penetration of EVs under neuroinflammation were attributed to interactions between the integrin lymphocyte function-associated antigen 1 (LFA1) on the EV surface, and intercellular adhesion molecule 1 (ICAM-1) overexpressed on the inflamed brain endothelium [45]. Finally, the use of the evolutionary conserved L-domain pathway was suggested for loading therapeutic proteins into EVs [122]. A target protein, Cre recombinase, was labeled with a specific WW tag that resulted in the specific interaction with L-domain containing protein Ndfip1 and packaging in EVs. The functional delivery of Cre recombinase in EVs to recipient cells was documented in transgenic mice upon intranasal administration [122]. Overall, the co-incubation method is relatively simple to scale up. Furthermore, this approach makes it possible to work with highly fragile therapeutic molecules, although it usually does not provide high loading efficiency of EVs nanocarriers.

#### 4.1.2. Sonication

Another promising method for loading small molecule drugs, as well as macromolecules, is sonication. Treatment with ultrasound produces transient pores in EV membranes that allow drug diffusion inside the vesicles. This method may provide higher loading efficiency compared to simple co-incubation. Two different approaches could be utilized: water bath sonication and probe sonication in the presence of the drug. Water bath sonication is a mild procedure that can be used when a therapeutic with relatively low stability needs to be formulated. For example, lysosomal enzyme, tripeptidyl peptidase-1 (TPP1) was loaded into EVs under sonication in a water bath [44]. Up to 70 µg TPP1 was loaded into 10^11^ EVs particles with enzymatic activity 44,000 mU/min by this method. Accordingly, EVs significantly increased the stability of TPP1 against protease degradation and provided efficient TPP1 delivery to target cells in in vitro and in vivo models of late-infantile neuronal ceroid lipofuscinosis (LINCL). A robust brain accumulation of EV carriers and increased lifespan was recorded in LINCL mice following the intraperitoneal administration of TPP1-loaded EVs [44]. Next, water bath sonication followed by extrusion was used to load Dox into EV-mimetic hybrid vesicles [97]. Lamichhane et al. [123] reported loading functional small interfering RNA (siRNA) and microRNA (miRNA) into EVs via sonication in a water bath. EVs loaded with siRNA retained their integrity and mediated knockdown of target genes, leading to cognate protein downregulation. Of note, the overall delivery of labeled siRNA to cells via sonicated EVs was low (2.96%), especially given that the cell uptake of labeled EVs was >80% [123]. These results corroborate those in a recent publication by Albanese et al. [124]. The authors were able to achieve a substantial binding of EVs to a range of different target cells in vitro, but failed to detect a fusion between EVs and recipient cells, and the subsequent release of the EV cargo (miRNAs) into the cytoplasm. This raises a very important question about the mechanism of delivery of the therapeutic cargo into targeted cells. Specifically, whether the direct membrane fusion of EVs with plasma membrane or membranes of endocytic vesicle followed by release of therapeutic cargo into the cytoplasm occurred upon EV internalization by recipient cells.

A probe sonication can provide higher loading efficiency, but should be used with relatively stable therapeutics, such as small molecule anticancer agents, Ptx and Dox, as reported in [111,112]. These drugs were chosen as model drugs for their hydrophobic and hydrophilic properties, respectively [112]. Optimization sonication procedures, along with pH, and temperature of EVs/drug mixture, resulted in high loading efficiency of macrophage derived EV formulations. Thus, using a pH that is close to pI for Dox (i.e., decreasing the charge of the drug molecule and increasing its hydrophobicity) significantly increased Dox loading into EVs. Another important finding was that the temperature of the mixture drastically affected the drug loading. Specifically, the sonication of EVs with Ptx at room temperature (RT) with short periods of cooling in between the cycles was more efficient that sonication on ice that was maintained during sonication and intermediate periods [112]. Finally, the systemic administration of EVs loaded with Ptx and Dox resulted in the potent tumor growth suppression in mice with triple negative breast cancer (TNBC) solids, as compared to Taxol or Dox treated animals. Next, the probe sonication method was also used to load Dox into pH-sensitive EVs with hyaluronic acid grafted with deoxycholic acid (HDEA) [113]. The incorporation of HDEA allowed the researchers to accelerate release of encapsulated Dox in cancer cells due to the acidic pH-induced protonation of 3-(diethylamino)propylamine (DEAP) moieties [113]. In another work, Li et al. [43] used the sonication method to load a small molecule drug, Gem. Notably, the sonication method provided higher loading efficiency compared to co-incubation; however, ultrasound treatment may result in the destruction and inactivation of some therapeutic molecules.

Beside the incorporation of small molecule therapeutics, probe sonication was utilized to load macromolecules, such as proteins, enzymes, and growth factors. For example, a potent antioxidant, catalase was loaded into macrophage-derived EVs via a sonication procedure [43]. A reformation of EVs upon sonication resulted in high loading efficiency (around 26.1 ± 1.2%), sustained release, and catalase preservation against proteases degradation. About 940 ± 15 catalase molecules per nanocarrier were incorporated by sonication. Of note, the intensity of sonication upon loading may affect the morphology of the aggregates. Thus, “strong sonication” resulted in the appearance nonspherical associates with a variety of shapes, indicating the destruction of the initial EV structure [43]. In contrast, “mild sonication” with intermediate brakes for mixture cooling ensured the preservation of the EV structure and morphology, along with the high loading efficiency. Each mg of EV-based formulation of catalase (exoCAT) obtained by sonication contained approximately 1,376 ± 64.1 U of catalase activity in 4 × 10^11^ EVs/mL. The systemic administration of exoCAT provided significant neuroprotective effects in mouse models of PD [43].

#### 4.1.3. Electroporation

Electroporation is widely used to load small molecule drugs and nucleic acids. Takenaka et al. [116] utilized this method of exogenous loading to improve the delivery of Dox to lung cancer cells. The efficiency of Dox encapsulation into EVs was calculated to be 0.5%, based on the quantification of Dox using a spectrofluorometer. The cytotoxicity of Dox incorporated into EVs was examined in lung cancer cells in vitro and in vivo and compared to Dox incorporated into artificial liposomes. Interestingly, cotreatment with gefitinib (Gef), a tyrosine kinase inhibitor of epidermal growth factor receptor (EGFR), resulted in significant increases in Dox cytotoxicity in EV-based formulations, but not in liposome-based formulation. The results indicated that Gef can enhance cell-to-cell communication via EVs within the tumor microenvironment that was not applicable for synthetic liposomal nanocarriers. Thus, in some cases, using EVs instead of synthetic nanocarriers may completely alter the mechanism of drug therapeutic activity.

The electroporation method is widely used for loading small molecule drugs and nucleic acids into EVs [72,96,114,125,126]. Alvarez-Erviti et al. [114] pioneered this method, electroporating siRNA into DC-derived EVs. To reduce immunogenicity, autologous dendritic cells were used for EV production. To target the formulation to the brain, parent DCs were engineered to express Lamp2b, an EVs membrane protein, fused to the neuron specific RVG peptide. Systemically administered RVG-EVs loaded with exogenous siRNA produced strong mRNA (60%) and protein (62%) knockdown of BACE1, a therapeutic target in Alzheimer’s disease. The electroporation technique was also used to incorporate Alexa Fluor 488-tagged siRNA against MAPK1 into plasma EVs [117]. Fluorescently labeled siRNA was detected in recipient monocytes and lymphocytes isolated from the peripheral blood of healthy donors following incubation with electroporated EVs. EV-delivered siRNA caused selective downregulation of MAPK1 in recipient cells. Later, the same method was used to load EVs with miRNA to EGFR-expressing breast cancer cells [115]. In a recent study, the electroporation method was utilized to load tumor suppressor miRNAs into fibroblast-derived EVs to treat ovarian cancer [120]. Repeated intraperitoneal injections of miRNA-loaded EVs drastically inhibited peritoneal dissemination and reduced the tumor burden in a xenograft ovarian cancer mouse model. The electroporation of engineered extracellular vesicles allowed 15–20% encapsulation efficiency of siRNA and resulted in effective gene silencing, i.e., comparable to that of commercial Lipofectamine RNAiMax [96]. This is especially important considering that large quantities of these engendered EVs were manufactured by membrane fusion with synthetic lipids. Of note, electroporation may result in the aggregation of miRNA and co-isolation with EVs preparation [127]. Therefore, efficient purification of drug loaded EVs from nonincorporated miRNA should be developed to obtain reliable and reproducible therapeutic effects.

#### 4.1.4. Transient Permeabilization with Saponin

To load therapeutic proteins such as catalase and TPP1, EV membranes were permeabilized with saponin [43,44]. For this purpose, EVs were first, pre-incubated with saponin to allow the selective removal membrane-bound cholesterol in the membranes, creating transient holes/pores in the EV bilayers, and thus, promoting drug loading (up to 6.3 µg/10^11^ particles for catalase, or 50 µg/10^11^ particles for TPP1 were incorporated). The successful delivery of these therapeutic enzymes to CNS manifested in the prevention of neurodegeneration and neuroinflammation in mouse models of Parkinson’s disease and a Lysosomal storage disorder, CLN2 [43,44]. Furthermore, modified calcium-mediated transfection led to the increased permeabilization and loading small RNAs into serum EVs [121].

#### 4.1.5. Freeze-Thaw and Extrusion Cycles

Finally, freeze-thaw cycles, and extrusion of EVs in the presence of a therapeutic protein were suggested for drug incorporation into macrophage-derived EVs [43]. A formation of large aggregates was reported during loading via freeze-thaw cycles, probably due to interaction via adhesive proteins on the surface of EVs. On the contrary, extrusion procedure led to the formation of relatively unisize aggregates with the efficient drug incorporation (22%). Nevertheless, almost 50% of EVs were lost via extrusion procedure when 200 nm pore size polycarbonate extruder membranes were used. Next, the effect of loading procedures (extrusion, freeze-drying, and probe sonication) on the structure, lipid and protein content, as well as interaction with target cells was extensively studied by Saux et al. [128]. The authors advocated for extrusion, as the most promising loading procedure compared to sonication and freeze-drying. Of note, most of these studies on the beneficial effects of EVs are preliminary studies in animal models and their translational potential has not yet been assessed in patients.

Overall, a mixture of exosomes and MVs is generally used for manufacturing EV-based drug formulations, as was mentioned above. However, some studies indicate that the accumulation and therefore, therapeutic effects of these two types of vehicles might differ. Thus, in observing the differences in unloading methods of EVs, Saari et al. [106] analyzed the pathways of Ptx uptake in both exosomes and MVs from the human prostate cancer cell line PC-3 cells utilizing fluorescence lifetime imaging microscopy (FLIM). Results concluded that the drug delivery pathways of exosomes and MVs differ, demonstrating that exosomes mainly deliver by endocytosis, while MVs discharges its therapeutic cargo by fusion with the cell membrane, as well as endocytosis. Thus, one might consider advantages of using homogeneous population of exosomes or MVs for drug incorporation vs. manufacture cost. Indeed, efficient purification of loaded EVs from nonincorporated drug is of great importance for the assessment of their role in therapeutic nanomedicine applications. The contamination of free cargo may mask or even mislead the overall conclusions about efficiency of EV-based drug formulations.

Furthermore, the location of the therapeutic cargo, specifically, where the drug is situated, i.e., on the luminal or abluminal site of EVs, or incorporated into membrane of the vesicle will determine the level of drug protection and in the blood circulation, kinetics of drug release, and overall immunogenicity of EV-based formulation. The unique structure of EVs made of a hydrophobic lipid bilayer and a hydrophilic cavity allows for a loading of different therapeutic agents that have hydrophobic or hydrophilic properties [129]. Hence, therapeutic molecules may diffuse inside the EVs or simply adhered to their surface. In many cases, the location of the cargo is controlled by its solubility in water. For example, Haney at al. [112] utilized two chemotherapeutic agents, Dox and Ptx, as representatives of hydrophilic and hydrophobic small molecule antineoplastic agents, respectively. Dox, a relatively hydrophilic compound would be incorporated into EVs inner compartment, while Ptx, a highly hydrophobic compound, probably resided in the membranes of the EVs. The thorough characterization is crucial for the manufacture of reliable and reproducible EV-based formulations.

### 4.2. Exogenous Loading of Parent Cells

In exploring exogenous loading of parent cells, two methods are considered: the loading of therapeutic cargo into isolated parent cells, and the transfection of parent cells with therapeutics encoding plasmid DNA (*p*DNA). The main cases of different loading procedures are outlined in Table 2.

Regarding the loading of parent cells_with small molecule therapeutic agents, the cells can be incubated with the desired therapeutic agent, and then EVs with the incorporated drug are isolated from cell conditioned media [24]. Diaz Osterman et al. [130] utilized this method for loading of pancreatic adenocarcinoma cells (PANC-1) in vitro with Cur, which has potential anticancer and anti-inflammatory benefits. It was reported that Cur was detected in the lumen of the EVs. In another study, MSCs-derived EVs were loaded with Ptx by incubating the parent cells with the drug, and then isolating EVs [131]. MSCs were able to package and deliver active Ptx through their EVs, suggesting the possibility of using these cells as a factory to develop drugs with a higher cell-target specificity. The same loading procedure through parent cells (HepG2) was used to produce EVs carrying different anticancer agents: Ptx, Etoposide, Carboplatin, Irinotecan, Epirubicin, and Mitoxantrone [132]. The obtained EV-based drug formulations showed potent antiproliferative activity on human pancreatic cell line CFPAC-1 [132]. Loading parent cells with anticancer agents, Dox, Gentamicin, 5-Fluoracil, or Carboplatin was also proposed for manufacture monocyte-derived EVs [54]. This approach allows manufacturing relatively large batches of EV-based formulations. However, a high amount of the drug required for the loading into the cells that is also partially metabolized by parent cells during incubation diminish clinical potential of this method.

Regarding using macromolecular therapeutics for loading through parent cells, one should pay a special attention to preserve these drugs against degradation in host cells. Thus, our lab utilized this approach for loading macrophage-derived EVs with catalase [43,134]. For this purpose, first, catalase was incorporated into a polymer-based nanocontainer, and then loaded into parent cells. Importantly, the formulation design of this nanoparticle was different from the common approach for cell-free drug nanoformulation. Nanocarriers for cell-free delivery are typically size-restricted to avoid entrapment in monocytes, focusing on small size nanoparticles with a polyethylene-glycol (PEG) corona to perpetuate a stealth effect. On the contrary, the best nanoformulation for loading into parent cells had a relatively large size (*c.a.* 200 nm) that resulted in improved accumulation in macrophages, and drug reshuffling into EVs. The cross-linking of polymer-based nanoparticles with an excess of a nonbiodegradable linker ensured low cytotoxicity of nanoformulation and efficient catalase protection in the parent cells [43,133]. Released EVs loaded with catalase were efficiently accumulated in target cells and produced potent anti-inflammatory and neuroprotective effects in Parkinson’s disease mouse models. Maugeri at al. [140] reported loading mRNA encoding human erythropoietin (hEPO protein) into EVs through parent cells. The mRNA was incorporated into lipid-based nanoparticles (LNPs) to protect against degradation in the cells. hEPO-mRNA and the LNP components i.e., cationic ionizable lipids were accumulated in endosomes, and then incorporated into intraluminal vesicles of the late endosomes that were subsequently secreted into the extracellular environment as EVs. Systemically administered drug-loaded EVs delivered hEPO-mRNA to cells and produce human protein in mouse blood and major organs [140].

The second method of exogenous loading is the transfection of parent cells with *p*DNA encoding the desired therapeutic, and then isolation EVs from cell conditioned media. Mizrak et al. [135] utilized this technique for transduction of donor cells to isolate EVs that contained the therapeutic cytosine deaminase-uracil phosphoribosyltransferase (CD-UPRT) protein. The direct injection of drug-loaded EVs, as well as systemic therapy with 5-fluorouracil (5-FU), proved effective in the regression of glioblastomas. Of note, this approach allowed to deliver both the therapeutic mRNA and protein via EVs for cancer treatment. Transfection of macrophages cell line, RAW264.7 with miR-16 inhibitor using Lipofectamine 2000 was reported by Jang et al. [136]. The authors indicated that upregulated miR-16 can be transferred to tumor-associated macrophages via EVs and inhibit tumor associated macrophages (TAM) infiltration and M2 polarization, suppressing tumor growth in murine breast cancer model. Furthermore, EVs with incorporated chicken egg ovalbumin (OVA) were obtained by transfecting parent cells with plasmid that encodes fusion protein consisting of OVA and specific protein that is known to localize to EVs [137]. We also reported the development of two EV-based drug delivery system for catalase [138], and glial cell-line derived neurotropic factor (GDNF) [134]. For this purpose, parent cells, macrophages were genetically modified with *p*DNA encoding these therapeutic proteins and drug-loaded EVs were collected from cell concomitant media. We demonstrated that pre-transfected macrophages released EVs with encapsulated *p*DNA, mRNA and the encoded therapeutic protein [138]. Furthermore, EVs contained a considerable amount of the transcription factor, NF-κB, which is particularly involved in the utilized protein expression under CMV promoter. Accumulation of these EVs in the brain led to potent and sustained therapeutic effects in PD mouse models. EVs efficiently transferred their contents to contiguous neurons resulting in *de novo* protein synthesis in target cells. Thus, EVs represent a highly efficient packaging system that can be used for the delivery of proteins and genetic material to target cells. An interesting approach for incorporation of adeno-associated virus (AAV) capsids into EVs to diminish their immunogenicity and improve gene delivery was suggested by Maguire et al. [139]. It was reported that during production, a fraction of released AAV vectors were associated with EVs, termed vEVs (vector-EVs), which outperformed conventionally purified AAV vectors in transduction efficiency in vitro. Indeed, EVs isolation method from parent cells conditioned media is crucial for apparent therapeutic activity and its interpretation. For example, coprecipitation of nucleic acids is common in many isolation procedures, especially PEG-based precipitation and ultracentrifugation [127,141]. Nevertheless, manufacturing pure drug loaded EVs formulations through parental cells may be more feasible than through loading of naive EVs with considerable amount of nonincorporated drug in the media.

## 5. Challenges of Insufficient Targeting Efficiency of EVs Formulations to Disease Tissues

One of the bottlenecks known for EV-based formulations is insufficient targeting capability. Recent biodistribution studies of unmodified EVs after intravenous injection revealed a rapid accumulation of EVs in organs of the reticuloendothelial system (RES), such as the liver, kidney and spleen after systemic administration [142,143]. In this context, the need for EVs with enhanced targeting efficiencies is crucial. The addition of targeting moieties to EVs nanocarriers can be accomplished through their incorporation into naive EVs, or through genetic modification of parent cells. Regarding incorporation of vector moiety into naive EVs or parent cells, the molecule should consist of lipid-like part for efficient integration into EVs membranes and a targeting fragment. For example, anisamide (AA) vector moiety was used to target EVs to the sigma receptor overexpressed on many cancer cells lung cancer cells [111]. The efficient encoring of AA into EVs lipid bilayers was accomplished by using AA conjugated with lipid moiety, 1,2-distearoryl-sn-glycero-3-phospho ethanolamine-*N*-[methoxy(polyethyleneglycol-2000) (DSPE-PEG) [111]. The EVs vectorization was accomplished simultaneously upon Ptx loading via probe sonication of the EVs, Ptx, and DSPE-PEG-AA mixture. A profound ability to accumulate in cancer cells upon systemic administration and eradicate pulmonary metastases were demonstrated in mouse model of pulmonary metastases with Lewis Lung Carcinoma cells [111]. Wan et al. [144] reported vectorization EVs to cancer cells via incorporation of lipidated ligand, a nucleolin-targeting aptamer AS1411, that was covalently conjugated to cholesterol-poly(ethylene glycol) (cholesterol-PEG). The incorporation was achieved via mechanical extrusion of parent cells grafted with these target moieties. The vectorized EVs were then loaded with Ptx by probe sonication. The aptamer grafted EVs successfully delivered their therapeutic cargo Ptx in vitro and in vivo. Cell-type tropism is also an important factor in the achievement of tumor cell targeting [145]. Conjugation of BBB-targeting cyclo(Arg-Gly-Asp-D-Tyr-Lys) peptide, c(RGDyK), to the EVs surface by the copper-free click chemistry was reported by Tian et al. [146]. Specifically, this peptide is known to exhibit high affinity to integrin αvβ3 in reactive cerebral vascular endothelial cells after ischemia [147]. Systemic administration of BBB-targeted c(RGDyK)-EVs loaded with antioxidant curcumin (Cur) resulted in a strong suppression of the inflammatory response and cellular apoptosis in the lesion region in the transient middle cerebral artery occlusion (MCAO) mice model.

The second approach is widely used for targeting EVs through genetic modification of parent cells. In recent work, Alvarez-Erviti et al. vectorized EVs to the brain by transfecting parent dendritic cells (DCs) with plasmid encoding fusion protein consisting of lysosomal-associated membrane protein 2 (Lamp2b) and neuron-specific peptide derived from rabies virus glycoprotein (RVG) [114]. It was reported that RVG-vectorized EVs loaded with glyceraldehyde 3-phosphate dehydrogenase (GAPDH) siRNA successfully delivered the therapeutic siRNA in wild-type mice to the cells of neurovascular unit and caused efficient knock down of BACE1, which represents a therapeutic target in Alzheimer’s disease [114]. Ishikawa et al. [148] reported vectorization of EVs to cancer tissues with programmed cell death ligand 1 (PD-L1). To modify EVs surface, the insect cell line Spodoptera frugiperda 9 was infected with recombinant baculoviruses incorporating the PD-1 mutant gene to express the target membrane protein. Engineered EVs expressing the high-affinity PD-1 mutants effectively bound PD-L1 and PD-L1-expressing cancer cells, showing potential as a candidate in new therapy approaches.

## 6. EV-Based Drug Formulations in Clinic

An increasing amount of research effort has focused on moving EV-based formulations into the clinic. Currently, www.clinicaltrials.gov reports about 182 studies involving EVs as interventions or as a study object. Most clinical studies are concentrated on the use of EV as markers for diseases prognosis and diagnosis. Twenty-three clinical trials are focused on extracellular vesicles as a therapeutics or drug delivery agents (Table 3).

A large-scale production EV-based formulation is critical for success of clinical translation. As of today, the good manufacturing procedures (GMP) have been developed in Industry and Academia [149,150,151]. For example, a group in Wake Forest Institute for Regenerative Medicine labs has developed formulation of EVs loaded with therapeutic microRNAs. Furthermore, more than half a dozen clinical trials utilized EVs that proved feasibility and short-term safety of EVs formulations [152]. Thus, MolecuVax, Inc. developed EVs-based technology for delivery therapeutic peptides. Jazz Pharmaceutics Inc. is working on chemotherapeutics loaded into EVs to treat cancer. Finally, several companied manufactured drug-loaded EVs for treatment of neurodegenerative disorders (Stem cell Medicine Ltd. and Evox Therapeutics). In this case, it is very important that EVs formulations can be concentrated, lyophilized, and reconstituted in aqueous solutions without significant altering their properties [43].

The cellular origin for EVs varies from dendritic cells [153,154,155,156], reticulocytes [157], erythrocytes [158], monocytes [159], macrophages [160] to mesenchymal [161,162,163] and human iPSCs [164,165]. MSCs are the main source for EVs production for clinical trials. It has been reported that MSC-derived EVs can be easily scalable and safe for patients [166,167]. Phase I/II trial of intravenous administered of allogeneic mesenchymal stem cells derived EVs loaded with miR-124 in patients with chronic stroke (NCT03384433) found that dose up to 1.5 million cells/kg body weight was safe for patients (*N* = 15) and showed behavior end points improvement over 12 month [168]. EVs purified from monocyte cultures from 15 patients were used to treat advanced metastatic melanoma. The GMP process allowed harvesting about 5 × 10^14^ EVs MHC class II carriers [169]. In another trial, a total of 40 patients with advanced colorectal cancer received up to 2 × 10^12^ EVs weekly for 4 weeks [170]. The therapies were safe, feasible, and efficient in induction of antigen-specific T lymphocyte response. Phase II clinical trial of vaccination with tumor antigen-loaded dendritic cell-derived EVs has been completed in 2018 by Gustave Roussy Center (France) (NCT01159288). The result showed an enhance antitumor immunity of NK cells in patients with advanced Non-Small Cell Lung Cancer [156].

Early Phase I trial NCT04270006 will use adipose stem cells derived EVs isolated from the patient and injections will be carried out to the patient’s periodontal pockets to identify regenerative efficiency. Preclinical in vivo results revealed significant regenerative effect compared to adipose stem cells and conventional treatment [171]. Effect of MSC-derived EVs on dry eye symptom in patients with chronic graft versus host diseases (NCT04213248) will be identified in Phase I/II clinical trial (*N* = 27). Patients will receive 10 µg/drop vesicles as an eye drop four times/day for 14 days and effect will be monitored during the 12 weeks of follow-up. Earlier study reported that EVs treatment can inhibit activation and infiltration of CD4 T cells [172]. Phase I clinical trial led by Dr. Gauri Varadhachary (NCT03608631) will identify dose and potential side effects of mesenchymal stromal cell-0derived EVs loaded with KrasG12D siRNA in treatment of pancreatic cancer with KrasG12D mutation. Thus, 28 participants will receive EVs systemically over 15-20 minutes on days 1, 4, and 10. Treatment repeats every 14 days for up to 3 courses and patients will be monitored up to 1 year. Next, safety and efficiency of EVs administered by nasal drop in patients with Alzheimer disease will be evaluated in the Phase I clinical trial in Ruijin Hospital (China). Nine participants will get 5, 10 or 20 µg of product two times a week for 12 weeks. Liver and kidney function and treatment-related adverse events will be monitored for 12 weeks, and cognitive function of participants as well as quality of life evaluation will be performed at baseline and 48 weeks after the treatment. Topical application of EVs will be appraised in the patients with dystrophic epidermolysis bullosa (NCT04173650) and ulcer (NCT04134676). In both studies participants will receive treatments directly on wounds and response will be measured after 14 days.

Several clinical trials were recently added to examine efficiency and safety of EVs products against novel coronavirus (SARS-COVID-2). Aerosol inhalation of MSC-derived EVs 2 × 10^8^ vesicles/3 mL at Day 1, Day 2, Day 3, Day 4, Day 5 will be assessed in the clinical trial in Ruijin Hospital (China) (NCT04276987). Inhalation of two types of MSC EVs twice a day during 10 days with 0.5–2 × 10^10^ particles in 3 mL on 90 participants will be evaluated in Medical Centre Dynasty Samara (Russia) (NCT04491240). Bone-marrow derived EVs will be administered via infusion to 60 participants with severe COVID-19 symptoms (NCT04493242). All-cause mortality and median days to recovery will be monitored. Finally, Landmark Hospital (Naples, Florida) will recruit 20 patients with COVID-19 to deliver acellular, minimally manipulated vesicles derived from human amniotic fluid. Product will be administered intravenously with 2–5 × 10^11^ particles/mL in addition to the standard care to evaluate safety, side effects and treatment competence (NCT04384445). Safety and efficacy assessment will be observed in Phase I clinical trial led by Mustafa Cetin (NCT04389385). Donor originated COVID-19 specific T-cells derived EVs will be delivered to the patients with COVID-19 via inhalation. Treatment will be applied daily at the dose 2 × 10^8^ particles / 3 mL for 5 days.

## 7. Conclusions

EV-based therapeutics represent a next generation drug delivery system that could be a valuable tool for the therapy of various diseases. The advantages of these systems include ability to cross biological barriers, improve drug pharmacokinetics, increase therapeutic efficacy, and reduce toxic side effects commonly associated with the conventional synthetic nanocarriers. Furthermore, EVs nanocarriers can be chemically modified to incorporate various ligands for targeted drug delivery. Moreover, EVs carry multiple biological molecules which reflect their origin, making them a promising tool for assisting clinical diagnoses and evaluations of disease prognosis. Indeed, some technological, functional and safety features are still to be addressed. Nevertheless, the unprecedented level of attention and amount of work focused the development of EV-based formulations are grounds for optimism for their fast and extensive application in the clinic.

## Figures and Tables

**Table 1:** Summary of preclinical investigations with EV-based drug formulations obtained via exogenous loading.

Disease	Source of EVs	Therapeutic Cargo/Drug	Loading Method	Ref.
Inflammatory-related diseases	Neutrophil-derived EVs	Piceatannol	Co-incubation	[95]
Cancer	Bioinspired EV-mimetic nanovesicles	Doxorubicin	Co-incubation	[54]
Inflammatory-related diseases	EL-4 Cells	Curcumin	Co-incubation	[41]
Brain inflammatory-related diseases	Brain inflammation disease cells	Curcumin	Co-incubation	[102]
Cerebral diseases	Endothelial cells	Curcumin	Co-incubation	[103]
Cancer	Immature dendritic cells	Doxorubicin	Co-incubation	[104]
Prostate Cancer	PC-3 cells	Paclitaxel	Co-incubation	[106]
Parkinson’s Disease	Blood	Dopamine	Co-incubation	[108]
Melanoma	Me 30966	Acridine Orange	Co-incubation	[110]
Cardiotoxicity	Liposomes	Doxorubicin	Co-incubation	[119]
Brain inflammatory-related diseases	Macrophages	Brain derived neurotrophic factor (BDNF)	Co-incubation	[45]
Pancreatic Cancer	Autologous EVs	Gemcitabine	Co-incubation and probe sonification	[109]
Triple Negative Breast Cancer	Macrophages	Paclitaxel and Doxorubicin	Probe sonification	[112]
Cancer	BT-474 tumor cells/SK-N-MC tumor cells	Doxorubicin	Probe sonification	[113]
MDR Cancer	Macrophages	Paclitaxel	Sonification	[107]
Cancer	Hybrid EVs (EVs from macrophage hybridized with synthetic liposome)	Doxorubicin	Water bath sonification	[97]
Pulmonary metastases	Macrophages	Paclitaxel	Probe sonification and Electroporation	[111]
Brain-related diseases	Macrophages	TPP1	Water bath sonification and permeabilization with saponin	[44]
Brain-related diseases	DC-derived EVs	siRNA	Electroporation	[114]
Breast Cancer	EVs targeted to EGFR-expressing cells	miRNA	Electroporation	[115]
Lung Cancer	EVs	Doxorubicin	Electroporation	[116]
Ovarian Cancer	Fibroblasts-derived EVs	Tumor suppressor miRNA	Electroporation	[120]
Lung diseases	Serum-derived EVs	siRNA	Permeabilization with saponin	[121]
Parkinson’s Disease	EVs secreted by monocytes and macrophages	Catalase	Co-incubation, probe sonification, permeabilization with saponin, freeze-thaw cycles, and extrusion	[43]

**Table 2:** Summary of preclinical investigations with EV-based drug formulations obtained via exogenous loading.

Disease	Source of EVs	Therapeutic Cargo/Drug	Loading Method	Ref.
Cancer	Bioinspired EV-mimetic nanovesicles	Dox	Loading of parent cells with therapeutic agent	[54]
Parkinson’s disease	EVs secreted by monocytes and macrophages	Catalase	Loading of parent cells with therapeutic agent	[43]
Pancreatic Cancer	PANC-1 cells	Curcumin	Loading of parent cells with therapeutic agent	[130]
Cancer	MSC-derived EVs	Paclitaxel	Loading of parent cells with therapeutic agent	[131]
Carcinoma	HepG2 cells	Paclitaxel, Etoposide, Carboplatin, Irinotecan, Epirubicin, Mitoxanthrone	Loading of parent cells with therapeutic agent	[132]
Neurodegene- rative diseases	Bone marrow-derived macrophages	Catalase	Loading of parent cells with therapeutic agent	[133]
Parkinson’s disease	GDNF-transfected macrophages	Catalase	Loading of parent cells with therapeutic agent and Transfection of parent cells	[134]
Glioblastomas	Isolated EVs from CD-UPRT-treated cells	Therapeutic CD-UPRT	Transfection of parent cells	[135]
Breast Cancer	Isolated EVs from EGCG-treated 4T1 cells	EGCG	Transfection of parent cells	[136]
Cancer	Isolated EVs from OVAC1C2-treated cells	OVAC1C2	Transfection of parent cells	[137]
Neurodegene-rative diseases	Transfected macrophages	Catalase	Transfection of parent cells	[138]
Adeno-associated virus	VEVs	AAV vectors	Transfection of parent cells	[139]

**Table 3:** Summary of clinical trials with EVs as an intervention (source: clinicaltrials.gov).

EVs Source	Condition	Drug	Administration Route	Dose Reported	Phase	Study Identifier
MSCs	Cerebrovascular disorders/stroke	miR-124	*i.v.*	200 µg protein	1/2	NCT03384433
MSCss	Alzheimer Disease	no	nasal drip	5 μg–20 μg	1/2	NCT04388982
MSCs	Periodontitis	no	local	not reported	early 1	NCT04270006
MSCs	Neuralgia	no	*i.v.* epineurally	45 mg, 15 mg	n/a	NCT04202783
MSCs	Depression	no	*i.v.*	21 million cells	n/a	NCT04202770
MSCs	Diabetes Mellitus Type 1	no	*i.v.*	1.2 × 10^10^ –1.51 × 10^10^ particles/kg	2/3	NCT02138331
MSCs	Chronic Ulcer	no	topical	not reported	1	NCT04134676
Plasma	Ulcer	no	topical	not reported	Early 1	NCT02565264
MSC	Dystrophic Epidermolysis Bullosa	no	topical	not reported	1/2	NCT04173650
MSCs	Multiple Organ Failure	no	*i.v.*	150 mg once a day for 14 times	n/a	NCT04356300
MSCs	Healthy	no	inhalation	2 × 10^8^–20 × 10^8^ particles/3 ml	1	NCT04313647
Plant	Colon Cancer	curcumin	oral	not reported	1	NCT01294072
Plant	Polycystic Ovary Syndrome	no	oral	not reported	n/a	NCT03493984
Grape	Head and Neck Cancer Oral Mucositis	no	oral	not reported	1	NCT01668849
DCs	Non Small Cell Lung Cancer	MHC class I- class II- cancer antigens	*i.v.*	53–2422 μg protein/injection	2	NCT01159288
MSCs	Pancreatic Adenocarcinoma	KRAS G12D siRNA	*i.v.*	days 1, 4, and 10 (dose not reported)	1	NCT03608631
MSCs	SARS-CoV-2 pneumonia	no	inhalation	2 × 10^8^ particles/3 mL	1	NCT04276987
MSCs	SARS-CoV-2 pneumonia	no	inhalation	0.5 × 10^10^–2 × 10^10^ particles/3 ml	1/2	NCT04491240
Bone marrow	SARS-CoV-2 pneumonia	no	*i.v.*	not reported	2	NCT04493242
Human amniotic fluid	SARS-CoV-2 pneumonia	no	*i.v.*	2 × 10^10^ –5 × 10^10^ particles	1/2	NCT04384445
MSCs	Dry Eye	no	eye drop	10 µg/drop	1/2	NCT04213248
MSCs	Macular Holes	no	drop	50 μg or 20 μg	early 1	NCT03437759
COVID-19 Specific T Cells	SARS-CoV-2 pneumonia	no	inhalation	2 × 10^18^ particles/3 mL	1	NCT04389385

## References

[1] Chong S.Y., Lee C.K., Huang C., Ou Y.H., Charles C.J., Richards A.M., Neupane Y.R., Pavon M.V., Zharkova O., Pastorin G. (2019). Extracellular Vesicles in Cardiovascular Diseases: Alternative Biomarker Sources, Therapeutic Agents, and Drug Delivery Carriers. Int. J. Mol. Sci..

[2] Boulanger C.M., Loyer X., Rautou P.E., Amabile N. (2017). Extracellular vesicles in coronary artery disease. Nat. Rev. Cardiol..

[3] Zamani P., Fereydouni N., Butler A.E., Navashenaq J.G., Sahebkar A. (2019). The therapeutic and diagnostic role of exosomes in cardiovascular diseases. Trends Cardiovasc. Med..

[4] Jia G., Sowers J.R. (2020). Targeting endothelial exosomes for the prevention of cardiovascular disease. Biochim. Biophys. Acta Mol. Basis Dis..

[5] Tikhomirov R., Donnell B.R., Catapano F., Faggian G., Gorelik J., Martelli F., Emanueli C. (2020). Exosomes: From Potential Culprits to New Therapeutic Promise in the Setting of Cardiac Fibrosis. Cells.

[6] Wei J., Hollabaugh C., Miller J., Geiger P.C., Flynn B.C. (2020). Molecular Cardioprotection and the Role of Exosomes: The Future Is Not Far Away. J. Cardiothorac. Vasc. Anesth..

[7] Ramasubramanian L., Kumar P., Wang A. (2019). Engineering Extracellular Vesicles as Nanotherapeutics for Regenerative Medicine. Biomolecules.

[8] Akbari A., Jabbari N., Sharifi R., Ahmadi M., Vahhabi A., Seyedzadeh S.J., Nawaz M., Szafert S., Mahmoodi M., Jabbari E. (2020). Free and hydrogel encapsulated exosome-based therapies in regenerative medicine. Life Sci..

[9] Kumar S., Zhi K., Mukherji A., Gerth K. (2020). Repurposing Antiviral Protease Inhibitors Using Extracellular Vesicles for Potential Therapy of COVID-19. Viruses.

[10] Bell B.M., Kirk I.D., Hiltbrunner S., Gabrielsson S., Bultema J.J. (2016). Designer exosomes as next-generation cancer immunotherapy. Nanomedicine.

[11] Moore C., Kosgodage U., Lange S., Inal J.M. (2017). The emerging role of exosome and microvesicle- (EMV-) based cancer therapeutics and immunotherapy. Int. J. Cancer.

[12] Masaoutis C., Mihailidou C., Tsourouflis G., Theocharis S. (2018). Exosomes in lung cancer diagnosis and treatment. From the translating research into future clinical practice. Biochimie.

[13] Liu C., Su C. (2019). Design strategies and application progress of therapeutic exosomes. Theranostics.

[14] Xie X., Wu H., Li M., Chen X., Xu X., Ni W., Lu C., Ni R., Bao B., Xiao M. (2019). Progress in the application of exosomes as therapeutic vectors in tumor-targeted therapy. Cytotherapy.

[15] D’Agnano I., Berardi A.C. (2020). Extracellular Vesicles, A Possible Theranostic Platform Strategy for Hepatocellular Carcinoma-An Overview. Cancers.

[16] Scavo M.P., Depalo N., Tutino V., de Nunzio V., Ingrosso C., Rizzi F., Notarnicola M., Curri M.L., Giannelli G. (2020). Exosomes for Diagnosis and Therapy in Gastrointestinal Cancers. Int. J. Mol. Sci..

[17] Chung I.M., Rajakumar G., Venkidasamy B., Subramanian U., Thiruvengadam M. (2020). Exosomes: Current use and future applications. Clin. Chim. Acta.

[18] Jurj A., Zanoaga O., Braicu C., Lazar V., Tomuleasa C., Irimie A., Berindan-Neagoe I. (2020). A Comprehensive Picture of Extracellular Vesicles and Their Contents. Molecular Transfer to Cancer Cells. Cancers.

[19] Tran P.H., Xiang D., Nguyen T.N., Tran T.T., Chen Q., Yin W., Zhang Y., Kong L., Duan A., Chen K. (2020). Aptamer-guided extracellular vesicle theranostics in oncology. Theranostics.

[20] Sousa C., Pereira I., Santos A.C., Carbone C., Kovacevic A.B., Silva A.M., Souto E.B. (2017). Targeting dendritic cells for the treatment of autoimmune disorders. Colloids Surf. B Biointerfaces.

[21] Ha D., Yang N., Nadithe V. (2016). Exosomes as therapeutic drug carriers and delivery vehicles across biological membranes: Current perspectives and future challenges. Acta Pharm. Sin. B.

[22] Vader P., Mol E.A., Pasterkamp G., Schiffelers R.M. (2016). Extracellular vesicles for drug delivery. Adv. Drug Deliv. Rev..

[23] Villa F., Quarto R., Tasso R. (2019). Extracellular Vesicles as Natural, Safe and Efficient Drug Delivery Systems. Pharmaceutics.

[24] Batrakova E.V., Kim M.S. (2015). Using exosomes, naturally-equipped nanocarriers, for drug delivery. J. Control. Release.

[25] Thery C., Amigorena S., Raposo G., Clayton A. (2006). Isolation and characterization of exosomes from cell culture supernatants and biological fluids. Curr. Protoc. Cell Biol..

[26] Thery C., Ostrowski M., Segura E. (2009). Membrane vesicles as conveyors of immune responses. Nat. Rev. Immunol..

[27] Witwer K.W., Thery C. (2019). Extracellular vesicles or exosomes? On primacy, precision, and popularity influencing a choice of nomenclature. J. Extracell. Vesicles.

[28] Liu D., Kou X., Chen C., Liu S., Liu Y., Yu W., Yu T., Yang R., Wang R., Zhou Y. (2018). Circulating apoptotic bodies maintain mesenchymal stem cell homeostasis and ameliorate osteopenia via transferring multiple cellular factors. Cell Res..

[29] Van der Pol E., Coumans F.A., Grootemaat A.E., Gardiner C., Sargent I.L., Harrison P., Sturk A., van Leeuwen T.G., Nieuwland R. (2014). Particle size distribution of exosomes and microvesicles determined by transmission electron microscopy, flow cytometry, nanoparticle tracking analysis, and resistive pulse sensing. J. Thromb. Haemost..

[30] Vlassov A.V., Magdaleno S., Setterquist R., Conrad R. (2012). Exosomes: Current knowledge of their composition, biological functions, and diagnostic and therapeutic potentials. Biochim. Biophys. Acta.

[31] Turturici G., Tinnirello R., Sconzo G., Geraci F. (2014). Extracellular membrane vesicles as a mechanism of cell-to-cell communication: Advantages and disadvantages. Am. J. Physiol. Cell Physiol..

[32] Aryani A., Denecke B. (2016). Exosomes as a Nanodelivery System: A Key to the Future of Neuromedicine?. Mol. Neurobiol..

[33] Anand S., Samuel M., Kumar S., Mathivanan S. (2019). Ticket to a bubble ride: Cargo sorting into exosomes and extracellular vesicles. Biochim. Biophys. Acta Proteins Proteom..

[34] Elsharkasy O.M., Nordin J.Z., Hagey D.W., de Jong O.G., Schiffelers R.M., Andaloussi S.E., Vader P. (2020). Extracellular vesicles as drug delivery systems: Why and how?. Adv. Drug Deliv. Rev..

[35] Tschuschke M., Kocherova I., Bryja A., Mozdziak P., Volponi A.A., Janowicz K., Sibiak R., Piotrowska-Kempisty H., Izycki D., Bukowska D. (2020). Inclusion Biogenesis, Methods of Isolation and Clinical Application of Human Cellular Exosomes. J. Clin. Med..

[36] Dai H.I., Vugmeyster Y., Mangal N. (2020). Characterizing Exposure-Response Relationship for Therapeutic Monoclonal Antibodies in Immuno-Oncology and Beyond: Challenges, Perspectives, and Prospects. Clin. Pharmacol. Ther..

[37] Matsumoto J., Stewart T., Sheng L., Li N., Bullock K., Song N., Shi M., Banks W.A., Zhang J. (2017). Transmission of alpha-synuclein-containing erythrocyte-derived extracellular vesicles across the blood-brain barrier via adsorptive mediated transcytosis: Another mechanism for initiation and progression of Parkinson’s disease?. Acta Neuropathol. Commun..

[38] Winston C.N., Goetzl E.J., Akers J.C., Carter B.S., Rockenstein E.M., Galasko D., Masliah E., Rissman R.A. (2016). Prediction of conversion from mild cognitive impairment to dementia with neuronally derived blood exosome protein profile. Alzheimers Dement. Diagn. Assess. Dis. Monit..

[39] Chen C.C., Liu L., Ma F., Wong C.W., Guo X.E., Chacko J.V., Farhoodi H.P., Zhang S.X., Zimak J., Segaliny A. (2016). Elucidation of Exosome Migration across the Blood-Brain Barrier Model In Vitro. Cell. Mol. Bioeng..

[40] Kuroda H., Tachikawa M., Yagi Y., Umetsu M., Nurdin A., Miyauchi E., Watanabe M., Uchida Y., Terasaki T. (2019). Cluster of Differentiation 46 Is the Major Receptor in Human Blood-Brain Barrier Endothelial Cells for Uptake of Exosomes Derived from Brain-Metastatic Melanoma Cells (SK-Mel-28). Mol. Pharm..

[41] Sun D., Zhuang X., Xiang X., Liu Y., Zhang S., Liu C., Barnes S., Grizzle W., Miller D., Zhang H.G. (2010). A novel nanoparticle drug delivery system: The anti-inflammatory activity of curcumin is enhanced when encapsulated in exosomes. Mol. Ther..

[42] Yang T., Martin P., Fogarty B., Brown A., Schurman K., Phipps R., Yin V.P., Lockman P., Bai S. (2015). Exosome delivered anticancer drugs across the blood-brain barrier for brain cancer therapy in Danio rerio. Pharm. Res..

[43] Haney M.J., Klyachko N.L., Zhao Y., Gupta R., Plotnikova E.G., He Z., Patel T., Piroyan A., Sokolsky M., Kabanov A.V. (2015). Exosomes as drug delivery vehicles for Parkinson’s disease therapy. J. Control. Release.

[44] Haney M.J., Klyachko N.L., Harrison E.B., Zhao Y., Kabanov A.V., Batrakova E.V. (2019). TPP1 Delivery to Lysosomes with Extracellular Vesicles and their Enhanced Brain Distribution in the Animal Model of Batten Disease. Adv. Healthc. Mater..

[45] Yuan D., Zhao Y., Banks W.A., Bullock K.M., Haney M., Batrakova E., Kabanov A.V. (2017). Macrophage exosomes as natural nanocarriers for protein delivery to inflamed brain. Biomaterials.

[46] Marcoux G., Duchez A.C., Cloutier N., Provost P., Nigrovic P.A., Boilard E. (2016). Revealing the diversity of extracellular vesicles using high-dimensional flow cytometry analyses. Sci. Rep..

[47] Van der Pol E., Boing A.N., Harrison P., Sturk A., Nieuwland R. (2012). Classification, functions, and clinical relevance of extracellular vesicles. Pharmacol. Rev..

[48] Al-Nedawi K., Meehan B., Micallef J., Lhotak V., May L., Guha A., Rak J. (2008). Intercellular transfer of the oncogenic receptor EGFRvIII by microvesicles derived from tumour cells. Nat. Cell Biol..

[49] Andre F., Schartz N.E., Movassagh M., Flament C., Pautier P., Morice P., Pomel C., Lhomme C., Escudier B., le Chevalier T. (2002). Malignant effusions and immunogenic tumour-derived exosomes. Lancet.

[50] Valadi H., Ekstrom K., Bossios A., Sjostrand M., Lee J.J., Lotvall J.O. (2007). Exosome-mediated transfer of mRNAs and microRNAs is a novel mechanism of genetic exchange between cells. Nat. Cell Biol..

[51] Li L., Li C., Wang S., Wang Z., Jiang J., Wang W., Li X., Chen J., Liu K., Li C. (2016). Exosomes Derived from Hypoxic Oral Squamous Cell Carcinoma Cells Deliver miR-21 to Normoxic Cells to Elicit a Prometastatic Phenotype. Cancer Res..

[52] Kogure T., Lin W.L., Yan I.K., Braconi C., Patel T. (2011). Intercellular nanovesicle-mediated microRNA transfer: A mechanism of environmental modulation of hepatocellular cancer cell growth. Hepatology.

[53] Cai J., Qiao B., Gao N., Lin N., He W. (2019). Oral squamous cell carcinoma-derived exosomes promote M2 subtype macrophage polarization mediated by exosome-enclosed miR-29a-3p. Am. J. Physiol. Cell Physiol..

[54] Jang S.C., Kim O.Y., Yoon C.M., Choi D.S., Roh T.Y., Park J., Nilsson J., Lotvall J., Kim Y.K., Gho Y.S. (2013). Bioinspired exosome-mimetic nanovesicles for targeted delivery of chemotherapeutics to malignant tumors. ACS Nano.

[55] Coffman L.G., Choi Y.J., McLean K., Allen B.L., di Magliano M.P., Buckanovich R.J. (2016). Human carcinoma-associated mesenchymal stem cells promote ovarian cancer chemotherapy resistance via a BMP4/HH signaling loop. Oncotarget.

[56] Melzer C., von der Ohe J., Hass R. (2018). Concise Review: Crosstalk of Mesenchymal Stroma/Stem-Like Cells with Cancer Cells Provides Therapeutic Potential. Stem Cells.

[57] Vakhshiteh F., Atyabi F., Ostad S.N. (2019). Mesenchymal stem cell exosomes: A two-edged sword in cancer therapy. Int. J. Nanomed..

[58] Qi J., Zhou Y., Jiao Z., Wang X., Zhao Y., Li Y., Chen H., Yang L., Zhu H., Li Y. (2017). Exosomes Derived from Human Bone Marrow Mesenchymal Stem Cells Promote Tumor Growth Through Hedgehog Signaling Pathway. Cell. Physiol. Biochem..

[59] Wu S., Ju G.Q., Du T., Zhu Y.J., Liu G.H. (2013). Microvesicles derived from human umbilical cord Wharton’s jelly mesenchymal stem cells attenuate bladder tumor cell growth in vitro and in vivo. PLoS ONE.

[60] Bruno S., Camussi G. (2013). Role of mesenchymal stem cell-derived microvesicles in tissue repair. Pediatr. Nephrol..

[61] Bruno S., Collino F., Deregibus M.C., Grange C., Tetta C., Camussi G. (2013). Microvesicles derived from human bone marrow mesenchymal stem cells inhibit tumor growth. Stem Cells Dev..

[62] Takahara K., Ii M., Inamoto T., Nakagawa T., Ibuki N., Yoshikawa Y., Tsujino T., Uchimoto T., Saito K., Takai T. (2016). microRNA-145 Mediates the Inhibitory Effect of Adipose Tissue-Derived Stromal Cells on Prostate Cancer. Stem Cells Dev..

[63] Roccaro A.M., Sacco A., Maiso P., Azab A.K., Tai Y.T., Reagan M., Azab F., Flores L.M., Campigotto F., Weller E. (2013). BM mesenchymal stromal cell-derived exosomes facilitate multiple myeloma progression. J. Clin. Investig..

[64] Lee C., Mitsialis S.A., Aslam M., Vitali S.H., Vergadi E., Konstantinou G., Sdrimas K., Fernandez-Gonzalez A., Kourembanas S. (2012). Exosomes mediate the cytoprotective action of mesenchymal stromal cells on hypoxia-induced pulmonary hypertension. Circulation.

[65] Lai R.C., Yeo R.W., Tan K.H., Lim S.K. (2013). Exosomes for drug delivery—A novel application for the mesenchymal stem cell. Biotechnol. Adv..

[66] Arslan F., Lai R.C., Smeets M.B., Akeroyd L., Choo A., Aguor E.N., Timmers L., van Rijen H.V., Doevendans P.A., Pasterkamp G. (2013). Mesenchymal stem cell-derived exosomes increase ATP levels, decrease oxidative stress and activate PI3K/Akt pathway to enhance myocardial viability and prevent adverse remodeling after myocardial ischemia/reperfusion injury. Stem Cell Res..

[67] Katsuda T., Tsuchiya R., Kosaka N., Yoshioka Y., Takagaki K., Oki K., Takeshita F., Sakai Y., Kuroda M., Ochiya T. (2013). Human adipose tissue-derived mesenchymal stem cells secrete functional neprilysin-bound exosomes. Sci. Rep..

[68] Xin H., Li Y., Chopp M. (2014). Exosomes/miRNAs as mediating cell-based therapy of stroke. Front. Cell. Neurosci..

[69] Ilmer M., Vykoukal J., Boiles A.R., Coleman M., Alt E. (2014). Two sides of the same coin: Stem cells in cancer and regenerative medicine. FASEB J..

[70] Emanueli C., Shearn A.I., Angelini G.D., Sahoo S. (2015). Exosomes and exosomal miRNAs in cardiovascular protection and repair. Vascul. Pharmacol..

[71] Kang K., Ma R., Cai W., Huang W., Paul C., Liang J., Wang Y., Zhao T., Kim H.W., Xu M. (2015). Exosomes Secreted from CXCR4 Overexpressing Mesenchymal Stem Cells Promote Cardioprotection via Akt Signaling Pathway following Myocardial Infarction. Stem Cells Int..

[72] Lee Y., el Andaloussi S., Wood M.J. (2012). Exosomes and microvesicles: Extracellular vesicles for genetic information transfer and gene therapy. Hum. Mol. Genet..

[73] Ibrahim A.G., Cheng K., Marban E. (2014). Exosomes as critical agents of cardiac regeneration triggered by cell therapy. Stem Cell Rep..

[74] Jeyaseelan K., Lim K.Y., Armugam A. (2008). MicroRNA expression in the blood and brain of rats subjected to transient focal ischemia by middle cerebral artery occlusion. Stroke.

[75] Liu F.J., Lim K.Y., Kaur P., Sepramaniam S., Armugam A., Wong P.T., Jeyaseelan K. (2013). microRNAs Involved in Regulating Spontaneous Recovery in Embolic Stroke Model. PLoS ONE.

[76] Lusardi T.A., Murphy S.J., Phillips J.I., Chen Y., Davis C.M., Young J.M., Thompson S.J., Saugstad J.A. (2014). MicroRNA responses to focal cerebral ischemia in male and female mouse brain. Front. Mol. Neurosci..

[77] Zhang G., Zhu Z., Wang H., Yu Y., Chen W., Waqas A., Wang Y., Chen L. (2020). Exosomes derived from human neural stem cells stimulated by interferon gamma improve therapeutic ability in ischemic stroke model. J. Adv. Res..

[78] Bian B., Zhao C., He X., Gong Y., Ren C., Ge L., Zeng Y., Li Q., Chen M., Weng C. (2020). Exosomes derived from neural progenitor cells preserve photoreceptors during retinal degeneration by inactivating microglia. J. Extracell. Vesicles.

[79] Batrakova E.V., Kim M.S. (2016). Development and regulation of exosome-based therapy products. Wiley Interdiscip. Rev. Nanomed. Nanobiotechnol..

[80] Witwer K.W., Buzás E.I., Bemis L.T., Bora A., Lässer C., Lötvall J., Nolte-‘t Hoen E.N., Piper M.G., Sivaraman S., Skog J. (2013). Standardization of sample collection, isolation and analysis methods in extracellular vesicle research. J. Extracell. Vesicles.

[81] Tauro B.J., Greening D.W., Mathias R.A., Ji H., Mathivanan S., Scott A.M., Simpson R.J. (2012). Comparison of ultracentrifugation, density gradient separation, and immunoaffinity capture methods for isolating human colon cancer cell line LIM1863-derived exosomes. Methods.

[82] Taylor D.D., Shah S. (2015). Methods of isolating extracellular vesicles impact down-stream analyses of their cargoes. Methods.

[83] Taylor D.D., Zacharias W., Gercel-Taylor C. (2011). Exosome isolation for proteomic analyses and RNA profiling. Methods Mol. Biol..

[84] Patel G.K., Khan M.A., Zubair H., Srivastava S.K., Khushman M., Singh S., Singh A.P. (2019). Comparative analysis of exosome isolation methods using culture supernatant for optimum yield, purity and downstream applications. Sci. Rep..

[85] Xu R., Greening D.W., Zhu H.J., Takahashi N., Simpson R.J. (2016). Extracellular vesicle isolation and characterization: Toward clinical application. J. Clin. Investig..

[86] Lener T., Gimona M., Aigner L., Borger V., Buzas E., Camussi G., Chaput N., Chatterjee D., Court F.A., del Portillo H.A. (2015). Applying extracellular vesicles based therapeutics in clinical trials—An ISEV position paper. J. Extracell. Vesicles.

[87] Chevillet J.R., Kang Q., Ruf I.K., Briggs H.A., Vojtech L.N., Hughes S.M., Cheng H.H., Arroyo J.D., Meredith E.K., Gallichotte E.N. (2014). Quantitative and stoichiometric analysis of the microRNA content of exosomes. Proc. Natl. Acad. Sci. USA.

[88] Moleirinho M.G., Silva R.J.S., Carrondo M.J.T., Alves P.M., Peixoto C. (2019). Exosome-based therapeutics: Purification using semi-continuous multicolumn chromatography. Sep. Purif. Technol..

[89] Navajas R., Corrales F.J., Paradela A. (2019). Serum Exosome Isolation by Size-Exclusion Chromatography for the Discovery and Validation of Preeclampsia-Associated Biomarkers. Methods Mol. Biol..

[90] Nordin J.Z., Lee Y., Vader P., Mager I., Johansson H.J., Heusermann W., Wiklander O.P., Hallbrink M., Seow Y., Bultema J.J. (2015). Ultrafiltration with size-exclusion liquid chromatography for high yield isolation of extracellular vesicles preserving intact biophysical and functional properties. Nanomedicine.

[91] Witwer K.W., van Balkom B.W.M., Bruno S., Choo A., Dominici M., Gimona M., Hill A.F., de Kleijn D., Koh M., Lai R.C. (2019). Defining mesenchymal stromal cell (MSC)-derived small extracellular vesicles for therapeutic applications. J. Extracell. Vesicles.

[92] Lamparski H.G., Metha-Damani A., Yao J.Y., Patel S., Hsu D.H., Ruegg C., Le Pecq J.B. (2002). Production and characterization of clinical grade exosomes derived from dendritic cells. J. Immunol. Methods.

[93] Ban J.J., Lee M., Im W., Kim M. (2015). Low pH increases the yield of exosome isolation. Biochem. Biophys. Res. Commun..

[94] Emam S.E., Ando H., Lila A.S.A., Shimizu T., Ukawa M., Okuhira K., Ishima Y., Mahdy M.A., Ghazy F.S., Ishida T. (2018). A Novel Strategy to Increase the Yield of Exosomes (Extracellular Vesicles) for an Expansion of Basic Research. Biol. Pharm. Bull..

[95] Gao J., Wang S., Wang Z. (2017). High yield, scalable and remotely drug-loaded neutrophil-derived extracellular vesicles (EVs) for anti-inflammation therapy. Biomaterials.

[96] Jhan Y.Y., Prasca-Chamorro D., Zuniga G.P., Moore D.M., Kumar S.A., Gaharwar A.K., Bishop C.J. (2020). Engineered extracellular vesicles with synthetic lipids via membrane fusion to establish efficient gene delivery. Int. J. Pharm..

[97] Rayamajhi S., Nguyen T.D.T., Marasini R., Aryal S. (2019). Macrophage-derived exosome-mimetic hybrid vesicles for tumor targeted drug delivery. Acta Biomater..

[98] De la Pena H., Madrigal J.A., Rusakiewicz S., Bencsik M., Cave G.W., Selman A., Rees R.C., Travers P.J., Dodi I.A. (2009). Artificial exosomes as tools for basic and clinical immunology. J. Immunol. Methods.

[99] Thone M.N., Kwon Y.J. (2020). Extracellular blebs: Artificially-induced extracellular vesicles for facile production and clinical translation. Methods.

[100] Li D., Yao S., Zhou Z., Shi J., Huang Z., Wu Z. (2020). Hyaluronan decoration of milk exosomes directs tumor-specific delivery of doxorubicin. Carbohydr. Res..

[101] Ju S., Mu J., Dokland T., Zhuang X., Wang Q., Jiang H., Xiang X., Deng Z.B., Wang B., Zhang L. (2013). Grape exosome-like nanoparticles induce intestinal stem cells and protect mice from DSS-induced colitis. Mol. Ther..

[102] Zhuang X., Xiang X., Grizzle W., Sun D., Zhang S., Axtell R.C., Ju S., Mu J., Zhang L., Steinman L. (2011). Treatment of brain inflammatory diseases by delivering exosome encapsulated anti-inflammatory drugs from the nasal region to the brain. Mol. Ther..

[103] Kalani A., Kamat P.K., Chaturvedi P., Tyagi S.C., Tyagi N. (2014). Curcumin-primed exosomes mitigate endothelial cell dysfunction during hyperhomocysteinemia. Life Sci..

[104] Tian Y., Li S., Song J., Ji T., Zhu M., Anderson G.J., Wei J., Nie G. (2014). A doxorubicin delivery platform using engineered natural membrane vesicle exosomes for targeted tumor therapy. Biomaterials.

[105] Rani S., Ryan A.E., Griffin M.D., Ritter T. (2015). Mesenchymal Stem Cell-derived Extracellular Vesicles: Toward Cell-free Therapeutic Applications. Mol. Ther..

[106] Saari H., Lisitsyna E., Rautaniemi K., Rojalin T., Niemi L., Nivaro O., Laaksonen T., Yliperttula M., Vuorimaa-Laukkanen E. (2018). FLIM reveals alternative EV-mediated cellular up-take pathways of paclitaxel. J Control. Release.

[107] Kim M.S., Haney M.J., Zhao Y., Mahajan V., Deygen I., Klyachko N.L., Inskoe E., Piroyan A., Sokolsky M., Okolie O. (2016). Development of exosome-encapsulated paclitaxel to overcome MDR in cancer cells. Nanomedicine.

[108] Qu M., Lin Q., Huang L., Fu Y., Wang L., He S., Fu Y., Yang S., Zhang Z., Zhang L. (2018). Dopamine-loaded blood exosomes targeted to brain for better treatment of Parkinson’s disease. J. Control. Release.

[109] Li Y.J., Wu J.Y., Wang J.M., Hu X.B., Cai J.X., Xiang D.X. (2020). Gemcitabine loaded autologous exosomes for effective and safe chemotherapy of pancreatic cancer. Acta Biomater..

[110] Iessi E., Logozzi M., Lugini L., Azzarito T., Federici C., Spugnini E.P., Mizzoni D., di Raimo R., Angelini D.F., Battistini L. (2017). Acridine Orange/exosomes increase the delivery and the effectiveness of Acridine Orange in human melanoma cells: A new prototype for theranostics of tumors. J. Enzyme Inhib. Med. Chem..

[111] Kim M.S., Haney M.J., Zhao Y., Yuan D., Deygen I., Klyachko N.L., Kabanov A.V., Batrakova E.V. (2018). Engineering macrophage-derived exosomes for targeted paclitaxel delivery to pulmonary metastases: In vitro and in vivo evaluations. Nanomedicine.

[112] Haney M.J., Zhao Y., Jin Y.S., Li S.M., Bago J.R., Klyachko N.L., Kabanov A.V., Batrakova E.V. (2019). Macrophage-Derived Extracellular Vesicles as Drug Delivery Systems for Triple Negative Breast Cancer (TNBC) Therapy. J. Neuroimmune Pharmacol..

[113] Park J., Lee H., Youn Y.S., Oh K.T., Lee E.S. (2020). Tumor-Homing pH-Sensitive Extracellular Vesicles for Targeting Heterogeneous Tumors. Pharmaceutics.

[114] Alvarez-Erviti L., Seow Y., Yin H., Betts C., Lakhal S., Wood M.J. (2011). Delivery of siRNA to the mouse brain by systemic injection of targeted exosomes. Nat. Biotechnol..

[115] Ohno S., Takanashi M., Sudo K., Ueda S., Ishikawa A., Matsuyama N., Fujita K., Mizutani T., Ohgi T., Ochiya T. (2013). Systemically injected exosomes targeted to EGFR deliver antitumor microRNA to breast cancer cells. Mol. Ther..

[116] Takenaka T., Nakai S., Katayama M., Hirano M., Ueno N., Noguchi K., Takatani-Nakase T., Fujii I., Kobayashi S.S., Nakase I. (2019). Effects of gefitinib treatment on cellular uptake of extracellular vesicles in EGFR-mutant non-small cell lung cancer cells. Int. J. Pharm..

[117] Wahlgren J., De L.K.T., Brisslert M., Sani F.V., Telemo E., Sunnerhagen P., Valadi H. (2012). Plasma exosomes can deliver exogenous short interfering RNA to monocytes and lymphocytes. Nucleic Acids Res..

[118] Hu L., Wickline S.A., Hood J.L. (2015). Magnetic resonance imaging of melanoma exosomes in lymph nodes. Magn. Reson. Med..

[119] Alyane M., Barratt G., Lahouel M. (2016). Remote loading of doxorubicin into liposomes by transmembrane pH gradient to reduce toxicity toward H9c2 cells. Saudi Pharm. J..

[120] Kobayashi M., Sawada K., Miyamoto M., Shimizu A., Yamamoto M., Kinose Y., Nakamura K., Kawano M., Kodama M., Hashimoto K. (2020). Exploring the potential of engineered exosomes as delivery systems for tumor-suppressor microRNA replacement therapy in ovarian cancer. Biochem. Biophys. Res. Commun..

[121] Zhang D., Lee H., Wang X., Rai A., Groot M., Jin Y. (2018). Exosome-Mediated Small RNA Delivery: A Novel Therapeutic Approach for Inflammatory Lung Responses. Mol. Ther..

[122] Sterzenbach U., Putz U., Low L.H., Silke J., Tan S.S., Howitt J. (2017). Engineered Exosomes as Vehicles for Biologically Active Proteins. Mol. Ther..

[123] Lamichhane T.N., Jeyaram A., Patel D.B., Parajuli B., Livingston N.K., Arumugasaamy N., Schardt J.S., Jay S.M. (2016). Oncogene Knockdown via Active Loading of Small RNAs into Extracellular Vesicles by Sonication. Cell. Mol. Bioeng..

[124] Albanese M., Chen Y.-F.A., Huls C., Gartner K., Tagawa T., Keppler O.T., Gobel C., Zeidler R., Hammerschmidt W. (2020). Micro RNAs are minor constituents of extracellular vesicles and are hardly delivered to target cells. BioRxiv.

[125] Al-Chalabi A., Jones A., Troakes C., King A., Al-Sarraj S., van den Berg L.H. (2012). The genetics and neuropathology of amyotrophic lateral sclerosis. Acta Neuropathol..

[126] El Andaloussi S., Lakhal S., Mager I., Wood M.J. (2013). Exosomes for targeted siRNA delivery across biological barriers. Adv. Drug Deliv. Rev..

[127] Kooijmans S.A.A., Stremersch S., Braeckmans K., de Smedt S.C., Hendrix A., Wood M.J.A., Schiffelers R.M., Raemdonck K., Vader P. (2013). Electroporation-induced siRNA precipitation obscures the efficiency of siRNA loading into extracellular vesicles. J. Control. Release.

[128] le Saux S., Aarrass H., Lai-Kee-Him J., Bron P., Armengaud J., Miotello G., Bertrand-Michel J., Dubois E., George S., Faklaris O. (2020). Post-production modifications of murine mesenchymal stem cell (mMSC) derived extracellular vesicles (EVs) and impact on their cellular interaction. Biomaterials.

[129] Villata S., Canta M., Cauda V. (2020). EVs and Bioengineering: From Cellular Products to Engineered Nanomachines. Int. J. Mol. Sci..

[130] Osterman C.J., Lynch J.C., Leaf P., Gonda A., Bennit H.R.F., Griffiths D., Wall N.R. (2015). Curcumin Modulates Pancreatic Adenocarcinoma Cell-Derived Exosomal Function. PLoS ONE.

[131] Pascucci L., Cocce V., Bonomi A., Ami D., Ceccarelli P., Ciusani E., Vigano L., Locatelli A., Sisto F., Doglia S.M. (2014). Paclitaxel is incorporated by mesenchymal stromal cells and released in exosomes that inhibit in vitro tumor growth: A new approach for drug delivery. J Control. Release.

[132] Lv L.H., Wan Y.L., Lin Y., Zhang W., Yang M., Li G.L., Lin H.M., Shang C.Z., Chen Y.J., Min J. (2012). Anticancer drugs cause release of exosomes with heat shock proteins from human hepatocellular carcinoma cells that elicit effective natural killer cell antitumor responses in vitro. J. Biol. Chem..

[133] Haney M.J., Suresh P., Zhao Y., Kanmogne G.D., Kadiu I., Sokolsky-Papkov M., Klyachko N.L., Mosley R.L., Kabanov A.V., Gendelman H.E. (2012). Blood-borne macrophage-neural cell interactions hitchhike on endosome networks for cell-based nanozyme brain delivery. Nanomedicine.

[134] Zhao Y., Haney M.J., Gupta R., Bohnsack J.P., He Z., Kabanov A.V., Batrakova E.V. (2014). GDNF-transfected macrophages produce potent neuroprotective effects in Parkinson’s disease mouse model. PLoS ONE.

[135] Mizrak A., Bolukbasi M.F., Ozdener G.B., Brenner G.J., Madlener S., Erkan E.P., Strobel T., Breakefield X.O., Saydam O. (2013). Genetically engineered microvesicles carrying suicide mRNA/protein inhibit schwannoma tumor growth. Mol. Ther..

[136] Jang J.Y., Lee J.K., Jeon Y.K., Kim C.W. (2013). Exosome derived from epigallocatechin gallate treated breast cancer cells suppresses tumor growth by inhibiting tumor-associated macrophage infiltration and M2 polarization. BMC Cancer.

[137] Zeelenberg I.S., Ostrowski M., Krumeich S., Bobrie A., Jancic C., Boissonnas A., Delcayre A., le Pecq J.B., Combadiere B., Amigorena S. (2008). Targeting tumor antigens to secreted membrane vesicles in vivo induces efficient antitumor immune responses. Cancer Res..

[138] Haney M.J., Zhao Y., Harrison E.B., Mahajan V., Ahmed S., He Z., Suresh P., Hingtgen S.D., Klyachko N.L., Mosley R.L. (2013). Specific transfection of inflamed brain by macrophages: A new therapeutic strategy for neurodegenerative diseases. PLoS ONE.

[139] Maguire C.A., Balaj L., Sivaraman S., Crommentuijn M.H., Ericsson M., Mincheva-Nilsson L., Baranov V., Gianni D., Tannous B.A., Sena-Esteves M. (2012). Microvesicle-associated AAV vector as a novel gene delivery system. Mol. Ther..

[140] Maugeri M., Nawaz M., Papadimitriou A., Angerfors A., Camponeschi A., Na M., Holtta M., Skantze P., Johansson S., Sundqvist M. (2019). Linkage between endosomal escape of LNP-mRNA and loading into EVs for transport to other cells. Nat. Commun..

[141] Mateescu B., Kowal E.J., van Balkom B.W., Bartel S., Bhattacharyya S.N., Buzas E.I., Buck A.H., de Candia P., Chow F.W., Das S. (2017). Obstacles and opportunities in the functional analysis of extracellular vesicle RNA—An ISEV position paper. J. Extracell. Vesicles.

[142] Lai C.P., Mardini O., Ericsson M., Prabhakar S., Maguire C., Chen J.W., Tannous B.A., Breakefield X.O. (2014). Dynamic biodistribution of extracellular vesicles in vivo using a multimodal imaging reporter. ACS Nano.

[143] Wiklander O.P., Nordin J.Z., O’Loughlin A., Gustafsson Y., Corso G., Mager I., Vader P., Lee Y., Sork H., Seow Y. (2015). Extracellular vesicle in vivo biodistribution is determined by cell source, route of administration and targeting. J. Extracell. Vesicles.

[144] Wan Y., Wang L., Zhu C., Zheng Q., Wang G., Tong J., Fang Y., Xia Y., Cheng G., He X. (2018). Aptamer-Conjugated Extracellular Nanovesicles for Targeted Drug Delivery. Cancer Res..

[145] Emam S.E., Lila A.S.A., Elsadek N.E., Ando H., Shimizu T., Okuhira K., Ishima Y., Mahdy M.A., Ghazy F.S., Ishida T. (2019). Cancer cell-type tropism is one of crucial determinants for the efficient systemic delivery of cancer cell-derived exosomes to tumor tissues. Eur. J. Pharm. Biopharm..

[146] Tian T., Zhang H.X., He C.P., Fan S., Zhu Y.L., Qi C., Huang N.P., Xiao Z.D., Lu Z.H., Tannous B.A. (2018). Surface functionalized exosomes as targeted drug delivery vehicles for cerebral ischemia therapy. Biomaterials.

[147] Guell K., Bix G.J. (2014). Brain endothelial cell specific integrins and ischemic stroke. Expert Rev. Neurother..

[148] Ishikawa R., Yoshida S., Sawada S.I., Sasaki Y., Akiyoshi K. (2020). Preparation of engineered extracellular vesicles with full-length functional PD-1 membrane proteins by baculovirus expression system. Biochem. Biophys. Res. Commun..

[149] Mendt M., Kamerkar S., Sugimoto H., McAndrews K.M., Wu C.C., Gagea M., Yang S., Blanko E.V.R., Peng Q., Ma X. (2018). Generation and testing of clinical-grade exosomes for pancreatic cancer. JCI Insight.

[150] Pachler K., Lener T., Streif D., Dunai Z.A., Desgeorges A., Feichtner M., Oller M., Schallmoser K., Rohde E., Gimona M. (2017). A Good Manufacturing Practice-grade standard protocol for exclusively human mesenchymal stromal cell-derived extracellular vesicles. Cytotherapy.

[151] Gimona M., Pachler K., Laner-Plamberger S., Schallmoser K., Rohde E. (2017). Manufacturing of Human Extracellular Vesicle-Based Therapeutics for Clinical Use. Int. J. Mol. Sci..

[152] Mentkowski K.I., Snitzer J.D., Rusnak S., Lang J.K. (2018). Therapeutic Potential of Engineered Extracellular Vesicles. AAPS J..

[153] Baghaei K., Tokhanbigli S., Asadzadeh H., Nmaki S., Zali M.R., Hashemi S.M. (2019). Exosomes as a novel cell-free therapeutic approach in gastrointestinal diseases. J. Cell. Physiol..

[154] Escudier B., Dorval T., Chaput N., Andre F., Caby M.P., Novault S., Flament C., Leboulaire C., Borg C., Amigorena S. (2005). Vaccination of metastatic melanoma patients with autologous dendritic cell (DC) derived-exosomes: Results of thefirst phase I clinical trial. J. Transl. Med..

[155] Morse M.A., Garst J., Osada T., Khan S., Hobeika A., Clay T.M., Valente N., Shreeniwas R., Sutton M.A., Delcayre A. (2005). A phase I study of dexosome immunotherapy in patients with advanced non-small cell lung cancer. J. Transl. Med..

[156] Besse B., Charrier M., Lapierre V., Dansin E., Lantz O., Planchard D., le Chevalier T., Livartoski A., Barlesi F., Laplanche A. (2016). Dendritic cell-derived exosomes as maintenance immunotherapy after first line chemotherapy in NSCLC. Oncoimmunology.

[157] Diaz-Varela M., de Menezes-Neto A., Perez-Zsolt D., Gamez-Valero A., Segui-Barber J., Izquierdo-Useros N., Martinez-Picado J., Fernandez-Becerra C., Del Portillo H.A. (2018). Proteomics study of human cord blood reticulocyte-derived exosomes. Sci. Rep..

[158] Hafiane A., Daskalopoulou S.S. (2018). Extracellular vesicles characteristics and emerging roles in atherosclerotic cardiovascular disease. Metabolism.

[159] Singhto N., Kanlaya R., Nilnumkhum A., Thongboonkerd V. (2018). Roles of Macrophage Exosomes in Immune Response to Calcium Oxalate Monohydrate Crystals. Front. Immunol..

[160] TRice F., Donaldson B., Bouqueau M., Kampmann B., Holder B. (2018). Macrophage-but not monocyte-derived extracellular vesicles induce placental pro-inflammatory responses. Placenta.

[161] Ferreira A.D.F., Gomes D.A. (2018). Stem Cell Extracellular Vesicles in Skin Repair. Bioengineering.

[162] de Godoy M.A., Saraiva L.M., de Carvalho L.R.P., Vasconcelos-Dos-Santos A., Beiral H.J.V., Ramos A.B., Silva L.R.P., Leal R.B., Monteiro V.H.S., Braga C.V. (2018). Mesenchymal stem cells and cell-derived extracellular vesicles protect hippocampal neurons from oxidative stress and synapse damage induced by amyloid-beta oligomers. J. Biol. Chem..

[163] Bobis-Wozowicz S., Kmiotek K., Kania K., Karnas E., Labedz-Maslowska A., Sekula M., Kedracka-Krok S., Kolcz J., Boruczkowski D., Madeja Z. (2017). Diverse impact of xeno-free conditions on biological and regenerative properties of hUC-MSCs and their extracellular vesicles. J. Mol. Med..

[164] Taheri B., Soleimani M., Aval S.F., Esmaeili E., Bazi Z., Zarghami N. (2019). Induced pluripotent stem cell-derived extracellular vesicles: A novel approach for cell-free regenerative medicine. J. Cell. Physiol..

[165] Adamiak M., Cheng G., Bobis-Wozowicz S., Zhao L., Kedracka-Krok S., Samanta A., Karnas E., Xuan Y.T., Skupien-Rabian B., Chen X. (2018). Induced Pluripotent Stem Cell (iPSC)-Derived Extracellular Vesicles Are Safer and More Effective for Cardiac Repair Than iPSCs. Circ. Res..

[166] Najar M., Bouhtit F., Melki R., Afif H., Hamal A., Fahmi H., Merimi M., Lagneaux L. (2019). Mesenchymal Stromal Cell-Based Therapy: New Perspectives and Challenges. J. Clin. Med..

[167] Bagno L., Hatzistergos K.E., Balkan W., Hare J.M. (2018). Mesenchymal Stem Cell-Based Therapy for Cardiovascular Disease: Progress and Challenges. Mol. Ther..

[168] Levy M.L., Crawford J.R., Dib N., Verkh L., Tankovich N., Cramer S.C. (2019). Phase I/II Study of Safety and Preliminary Efficacy of Intravenous Allogeneic Mesenchymal Stem Cells in Chronic Stroke. Stroke.

[169] Mignot G., Roux S., Thery C., Segura E., Zitvogel L. (2006). Prospects for exosomes in immunotherapy of cancer. J. Cell. Mol. Med..

[170] Dai S., Wei D., Wu Z., Zhou X., Wei X., Huang H., Li G. (2008). Phase I clinical trial of autologous ascites-derived exosomes combined with GM-CSF for colorectal cancer. Mol. Ther..

[171] Mohammed E., Khalil E., Sabry D. (2018). Effect of Adipose-Derived Stem Cells and Their Exo as Adjunctive Therapy to Nonsurgical Periodontal Treatment: A Histologic and Histomorphometric Study in Rats. Biomolecules.

[172] Lai P., Chen X., Guo L., Wang Y., Liu X., Liu Y., Zhou T., Huang T., Geng S., Luo C. (2018). A potent immunomodulatory role of exosomes derived from mesenchymal stromal cells in preventing cGVHD. J. Hematol. Oncol..

